# What Is the Right Chain Length? Liquid Crystalline Network Tuning by Molecular Design

**DOI:** 10.1021/acs.macromol.5c01011

**Published:** 2025-09-11

**Authors:** Simone Donato, Rachele Bini, Giovanni Simonetti, Neri Fuochi, Martina Salzano de Luna, Camille Chatard, Pierre-Louis Brient, Diederik S. Wiersma, Daniele Martella, Camilla Parmeggiani

**Affiliations:** † European Laboratory for Non-Linear Spectroscopy (LENS), via N. Carrara 1, Sesto Fiorentino 50019, Italy; ‡ Department of Physics and Astronomy − 9300 University of Florence, via G. Sansone 1, Sesto Fiorentino 50019, Italy; § Istituto Nazionale di Ricerca Metrologica (INRiM), Strada delle Cacce 91, Torino 10135, Italy; ∥ Department of Chemical, Materials and Industrial Production Engineering − 9307 University of Naples Federico II, Piazzale V. Tecchio, 80, Napoli 80125, Italy; ⊥ 585843 Specific Polymers, 150 Av. des Cocardières, Castries 34160, France; # Department of Chemistry “Ugo Schiff” − 9300 University of Florence, via della Lastruccia 3-13, Sesto Fiorentino 50019, Italy

## Abstract

Liquid crystalline networks (LCNs) are stimuli-responsive polymers with programmable actuation properties, including fast response times and tunable force generation. Their reversible deformations can be achieved under light irradiation when polymers are functionalized with photoresponsive molecules such as azobenzenes. All of these features need to be adapted for each specific application, and this is possible by tuning the material properties through molecular design. In this study, we demonstrate how to tailor both mechanical properties and light-dependent force development thanks to the synthesis of mesogenic cross-linkers with different alkyl chains. Acting on this molecular parameter allows modulating the maximum actuation force, while modifying the cross-linking degree is a more advantageous strategy to get fast activation. Our results provide valuable insights into the relationship between the molecular structure and material performance, paving the way for a rational design of innovative responsive materials.

## Graphical abstract

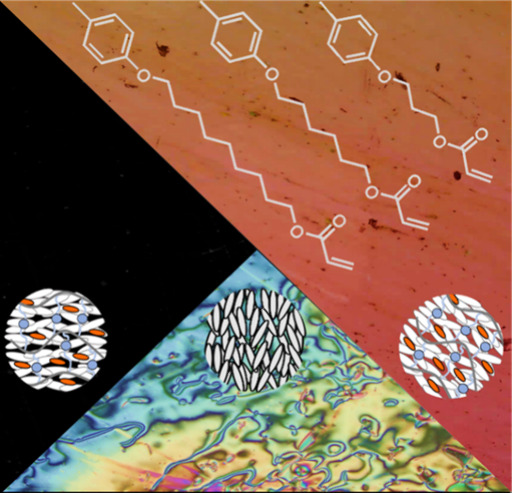

## Introduction

Liquid crystalline networks (LCNs) are very promising smart materials able to reversibly deform in response to external stimuli (including light and temperature) and they are currently employed for different applications, spanning from biomedical devices to soft robotics.^1–5^ These materials combine the elastic properties of cross-linked polymers with the responsiveness of liquid crystals (LCs). Their actuation is of great interest when light is used as an energy source since it offers non-invasive, remote control with high spatial resolution^6,7^ allowing for the development of miniaturized systems, including photonic devices and microrobots.^8–10^ To trigger the shape-change by light, the insertion of photoresponsive units, such as azobenzene moieties (undergoing photoinduced *cis*-*trans* isomerizations and/or photothermal dissipations), is needed toward fast (up to milliseconds) and reversible actuation.^11–13^

Regarding the preparation of LCNs, one of the most versatile techniques is the photopolymerization of aligned reactive mesogens, compatible with many alignment procedures (such as rubbed cells or photoalignment),^14–16^ and final designs (including coating, films, or 3D-printed micrometric objects).^17,18^ The material response is mainly driven by the monomer structure and their molecular alignment, and thus, understanding the relationship between these parameters and the actuator response is fundamental for the rational design of the final material. However, to date, this aspect is only partially tackled by empirical approaches,^19,20^ and most of the reported examples are based on a few commercial monomers, namely, RM257 and RM82, introduced by Broer et al. in 1989.^21^ These molecules have been extensively used in combination with a variety of chemical reactions and comonomers, but the lack of new research devoted to scalable synthesis of suitable monomers limits the knowledge on the specific role of the mesogen structure.

To take advantage of photopolymerization, mesogens – e.g. molecules exhibiting a LC phase in a certain temperature range^22^ – have to be decorated with polymerizable units. Once aligned and polymerized, the chaincross-linking allows the molecular order of the polymeric precursors to be preserved in the final material, resulting in the shape-changing behavior (Figure 1A). Indeed, the polymer contracts along a specific direction (the LC director) by the progressive loss of the nematic order upon heating (or other stimulation) until, in certain cases, a nematic to an isotropic phase transition. During cooling, the initial macroscopic shape (and molecular order) can be restored thanks to the cross-linked structures.^4,23–25^

**Figure 1:**
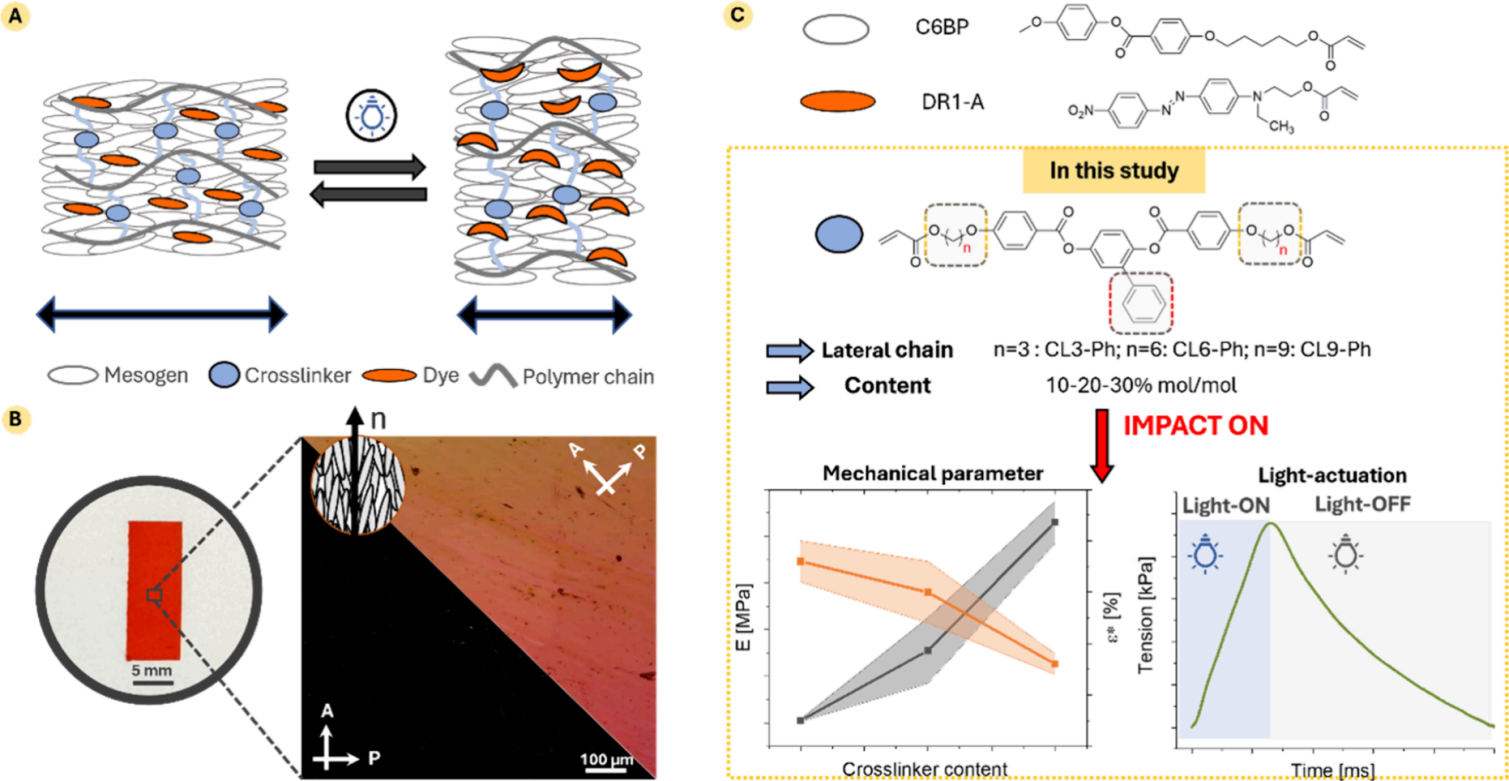
General behavior of LCNs. (A) Representation of the shape-changing behavior of azobenzene-doped LCNs. Deformation is induced by specific light irradiation; (B) exemplificative picture and POM images, highlighting the transmission changes by rotating the nematic director of the sample from 0° to 45° with respect to the crossed polarizer (P) and the analyzer (A); (C) molecular structure of the acrylate-based mesogens used in this study. The chemical modifications proposed strongly affect the mechanical properties and light-actuation behavior of the resulting LCNs.

Previous studies report on how specific chemical modification on mesogens affects the material transition temperatures,^26,27^ while different cross-linker amounts allow for the fine-tuning of its mechanical properties.^28^ For instance, increasing the cross-linking density improves the material mechanical strength, while using more flexible cross-linkers enhances LCN elasticity and responsiveness to external stimuli.^1,29^ In this framework, some of us recently demonstrated the presence of sterically hindered substituents in the cross-linker core to tune the thermal and mechanical behavior of the final LCNs, as well as its actuation performances, thanks to the destabilization of the LC core interactions.^26,30^

In this work, we further exploit the role of the chemical structure of the cross-linker in defining acrylate-based LCNs properties. Starting from a selected cross-linker mesogenic core (containing a lateral phenyl ring), chosen on the basis of our previous studies,^30^ we systematically characterize the role of the aliphatic chain length on the LCN behavior as artificial muscles. A major breakthrough of this study is the development of a wide adaptive and easily scalable synthetic protocol for mesogenic cross-linkers. These molecules have been tested for the LCN fabrication and, for the best performing composition, also the assessment of the effect of cross-linker concentration has been taken into account.

This comprehensive characterization reveals new insight into mechanical performance and activation dynamics, setting the stage for innovative applications in responsive and adaptive systems. The findings demonstrate how precise molecular design can unlock unprecedented capabilities of LCNs, with particular focus on their potential in next-generation artificial muscles and advanced actuation technologies.

## Experimental Section

### Material Preparation

C6BP (4-methoxybenzoic acid 4-(6-acryloyloxyhexyloxy) phenyl ester) was purchased from Synthon Chemicals; Irgacure 369 was purchased by Merck; cross-linkers and DR1-acrylate were synthesized by Specific Polymers as shown in the Supporting Information (Figure S1–S11). Composition of all of the monomeric mixtures is reported in Table S1. For film fabrication, the mixture was first heated to the isotropic phase and then infiltrated into a LC cell via capillarity. The LC cells were prepared by 2 glasses coated with PVA 5% aqueous solution and rubbed unidirectionally with a velvet cloth to promote homogeneous planar alignment. The material was then cooled to room temperature to achieve a monodomain alignment followed by photopolymerization at 25 °C for 10 min under UV irradiation (385 nm, M385LP1-C4 lamp, Thorlabs, 1.2 mW/cm^2^). A post-curing step, involving additional UV irradiation for 10 min at 45 °C, was subsequently applied.^18,30^

### Mesomorphic Characterization

The phase transition temperatures of the cross-linkers, mixtures, and LCNs were determined using a differential scanning calorimeter (DSC, TA Instruments Q-2000, Milan, Italy) under a nitrogen atmosphere, with heating and cooling rates of 20 °C/min. Polarized optical microscopy (POM) was performed by using a Zeiss Axio Observer A1 inverted microscope equipped with crossed polarizers, a Linkam PE120 hot stage, and an Axio camera to capture images of the LC textures.

### Mechanical Characterization

The mechanical properties were assessed using a Tensometer 2020 (Alpha Technologies) under quasi-static tensile testing conditions. The film specimens were mounted between two custom-designed clamps, and the upper crosshead was moved at a constant rate of 0.5 mm/min until the films failed. The force applied on the films was recorded by a load cell with a maximum capacity of 10 N and a precision of 0.02 N. Stress (σ = force/initial cross-sectional area of the specimen) and strain (ε = displacement/initial length of the film) were derived from the measured force and displacement values. The geometric dimensions of each tested sample were measured at three points, and the average values were used for the calculations.

### Light-Actuation Characterization

The force generated by the LCN films under irradiation was measured using a custom-built apparatus described previously.^30^ The setup consisted of a force transducer (WPI KG4, 0–50 mN range) to which the LCN strip was clamped on both sides to allow an isometric configuration. Each strip was irradiated from both sides using two Thorlabs M470L5 lamps Thorlabs. The activation was controlled by the LabVIEW program with a multichannel driver. The reported data were obtained under pulsed light irradiation provided with three different irradiation intensities: 2.3, 4.4, and 6.2 mW/mm^2^. The kinetic behavior, for both activation and relaxation, was determined by a fit in the linear region of the curve for the first 100 ms in each step. Thermal imaging was performed using a FLIR A400 camera equipped with a 320 × 240-pixel uncooled microbolometer detector. The surface temperature of the sample reported was an average of the acquired temperatures in a region of interest (ROI) confined to the dimensions of the sample.

## Results and Discussion

### Preparation and Mesomorphic Properties of the Materials

LCNs reported in this work present a homogeneous planar alignment in which the LC molecules are oriented along the nematic director “n”. Such a molecular alignment can be easily observed by Polarized Optical Microscopy (POM) as shown in Figure 1B. In this picture, the optical anisotropy of a well aligned LCN resulted in a transmission change during the sample rotation with respect to the crossed polarizers, having a maximum and minimum of transmission every 45° of rotation.^22^ A critical aspect to obtaining these well-aligned materials is to perform the polymerization reaction in the nematic phase of the monomeric mixture after its (monodomain) alignment.

Our formulations include a monoacrylate mesogen (C6BP), mainly determining the LC properties and the alignment of the LCNs, an acrylate-based azobenzene dye (Dispersed Red 1 acrylate, DR1 acrylate), inducing the material light responsiveness, and a LC cross-linker, primarily contributing to the mechanical properties of the final material (Figure 1C).^31,32^ LCNs have already shown great potential as smart materials for artificial muscles. However, the contraction behavior of the heart requires a precise balance between the mechanical properties and activation speed. Our molecular approach enables simultaneous tuning of both mechanical parameters and light-responsiveness, which is essential to bring such systems closer to realistic bioinspired actuation.

New cross-linkers have been synthesized starting from a mesogenic core with 3 aromatic rings and a phenyl lateral substituent in the central one. Such cross-linkers are called CLX-Ph where “X” is the number of carbon atoms in the alkyl chain spacer and “Ph” indicates a phenyl substituent (in contrast with commercially available RM monomers that present a methyl group in the same position). Accordingly, the monomer mixture and the final materials (which differ only for the cross-linker chosen and/or its percentage) are called LCNX-Ph_Z_ where “Z” is the mol/mol content of the cross-linker.

Cross-linkers have been prepared starting from benzoic acid derivatives (having a specific alkyl chain) by esterification with substituted-hydroquinones (Scheme 1). Specific features of this procedure include the following: no need of purification by chromatographic columns, easy adaptability to a wide range of new cross-linkers (e.g., with different alkyl chains and hydroquinones), and easy scalability. Details on the synthetic protocols for molecules CL3-Ph and CL9-Ph and their characterizations are reported in the Supporting Information (Figure S1–S8), while preparation of CL6-Ph was previously reported by some of us.^30^

**Scheme 1:**
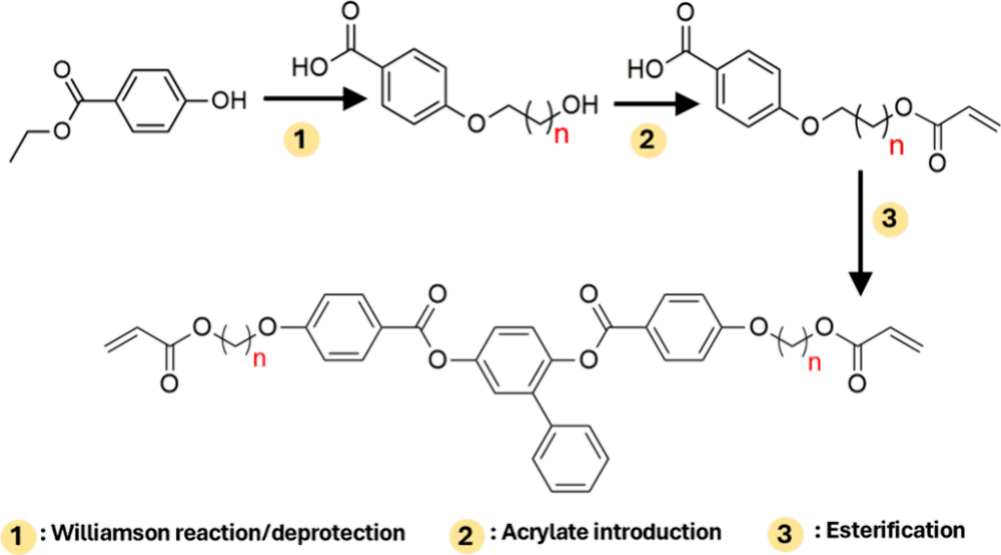
General Synthetic Steps for Reactive Mesogens

The mesomorphic properties of the molecules were evaluated through differential scanning calorimetry (DSC) and POM, and all the data are reported in the Supporting Information (Figure S9–S11). Cross-linker CL3-Ph did not exhibit any LC phase while, for the others, an isotropic-to-nematic transition was observed. The combination of the sterically hindered substituent on the central core (the Ph group) with the short aliphatic spacer in CL3-Ph results in an insufficient molecular flexibility, required to form liquid crystalline organization instead of crystallization, in contrast with commercial RM257 (having the same spacer but a methyl substituent in the core) that forms a nematic phase. As a consequence, after melting, CL3-Ph forms a crystal within a few minutes at room temperature, while the other cross-linkers remain in the nematic phase for many hours. The LC phase for both CL6-Ph and CL9-Ph has been attributed to nematic based on the Schlieren texture (with typical 2- or 4-branches defects) observed by POM.

Different monomer mixtures have been prepared using such cross-linkers in the amount of 10% mol/mol, while, only for CL6-Ph, higher amounts of diacrylate (20 and 30% mol/mol) have been also studied. The composition of each mixture is reported in Table S1. The phase transition temperatures of the monomeric mixtures are schematically represented in Figure 2A, while complete data are shown in Figures S12–S16 of the Supporting Information.

**Figure 2:**
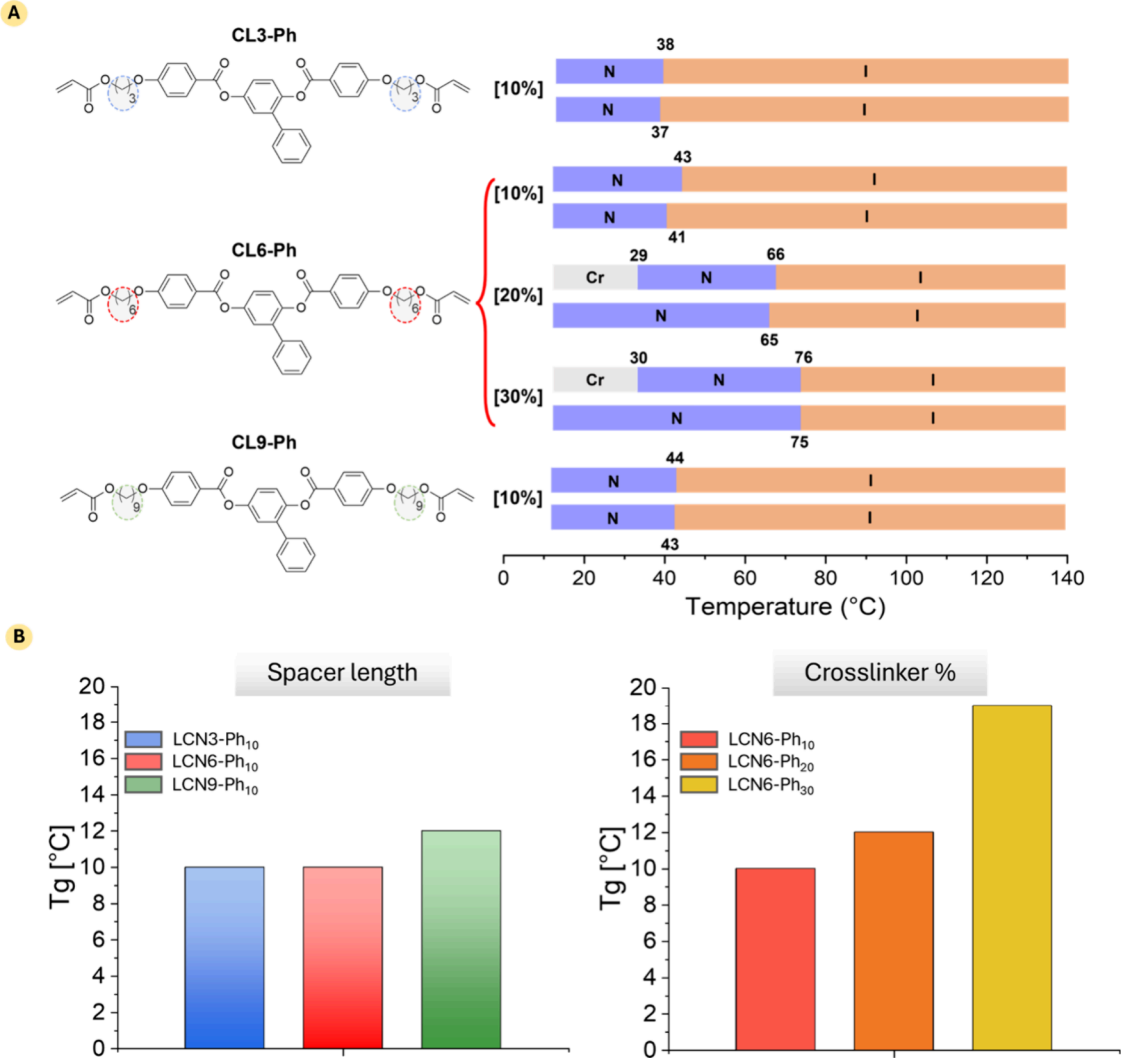
Thermal properties of the monomeric mixture and LCN films. (A) Monomer mixture phase transition temperatures chart. Temperatures are obtained during the second heating (top line)–cooling (bottom line) cycle of DSC experiments (20 °C min^–1^). Cr: crystalline phase; N: nematic phase; I: isotropic phase. All temperatures are taken at the maximum of the transition peak; (B) graphs reporting the glass transition temperatures of the final LCN films extrapolated from DSC traces. Heating cycle: 20 °C min^–1^.

After the first melting, all the mixtures showed a nematic phase transition in both heating and cooling with a negligible modification of the thermal behavior by changing the cross-linker structure. During the cooling stage, the formulations present a nematic phase at room temperature, highly desirable for the easy processing.^9,33^ Increasing the amount of CL6-Ph from 10 to 30% mol/mol, a cold-crystallization process was observed during the second heating cycle together with an increase of the clearing temperatures.

Then, LCN films were prepared in one-step by UV-photopolymerization (irradiation for 10 min at 25 °C and 10 min at 45 °C) of the monomer mixtures, allowing for the simultaneous growth of the polymeric chains and their cross-linking. This step has been performed after alignment of the monomeric mixture in an LC cell treated for the obtainment of the homogeneous planar alignment as widely described in previous works.^30^

The glass transition temperatures (*T*_g_) of the films, determined from the DSC traces (Figure S17), are reported in Figure 2B. The materials exhibited a rubbery state at room temperature, and no additional peaks were observed in the DSC traces. The analysis demonstrated how the elongation of the cross-linker spacer had a minor effect on the glass transition process, whereas a higher cross-linker content caused, as expected, a modest increase in the *T*_g_ from 10 to 19 °C.

### Mechanical Characterization of the LCN Films

A comprehensive mechanical characterization of the films was carried out by using a tensiometer to perform quasi-static tensile measurements. For each formulation, at least six replicates were tested on films with a thickness of about 50 μm, width of approximately 8 mm, and length of about 15 mm. During the tensile test, the films were stretched to failure, typically beginning with the formation of lateral cracks, which then propagated and eventually led to complete mechanical failure. The films were evaluated in two orientations – parallel and perpendicular to the nematic director – to understand how the anisotropic molecular arrangement of the material affects the mechanical behavior (Figure 3A). Previous studies demonstrate how the main mechanical parameters are strongly influenced by the LC alignment thus exhibiting a peculiar anisotropic mechanical behavior.^34,35^ Typically, Young’s modulus and stress at break are higher in the parallel direction than in the perpendicular one, while the elongation at break increases when the materials are tested perpendicularly to the alignment direction.

**Figure 3:**
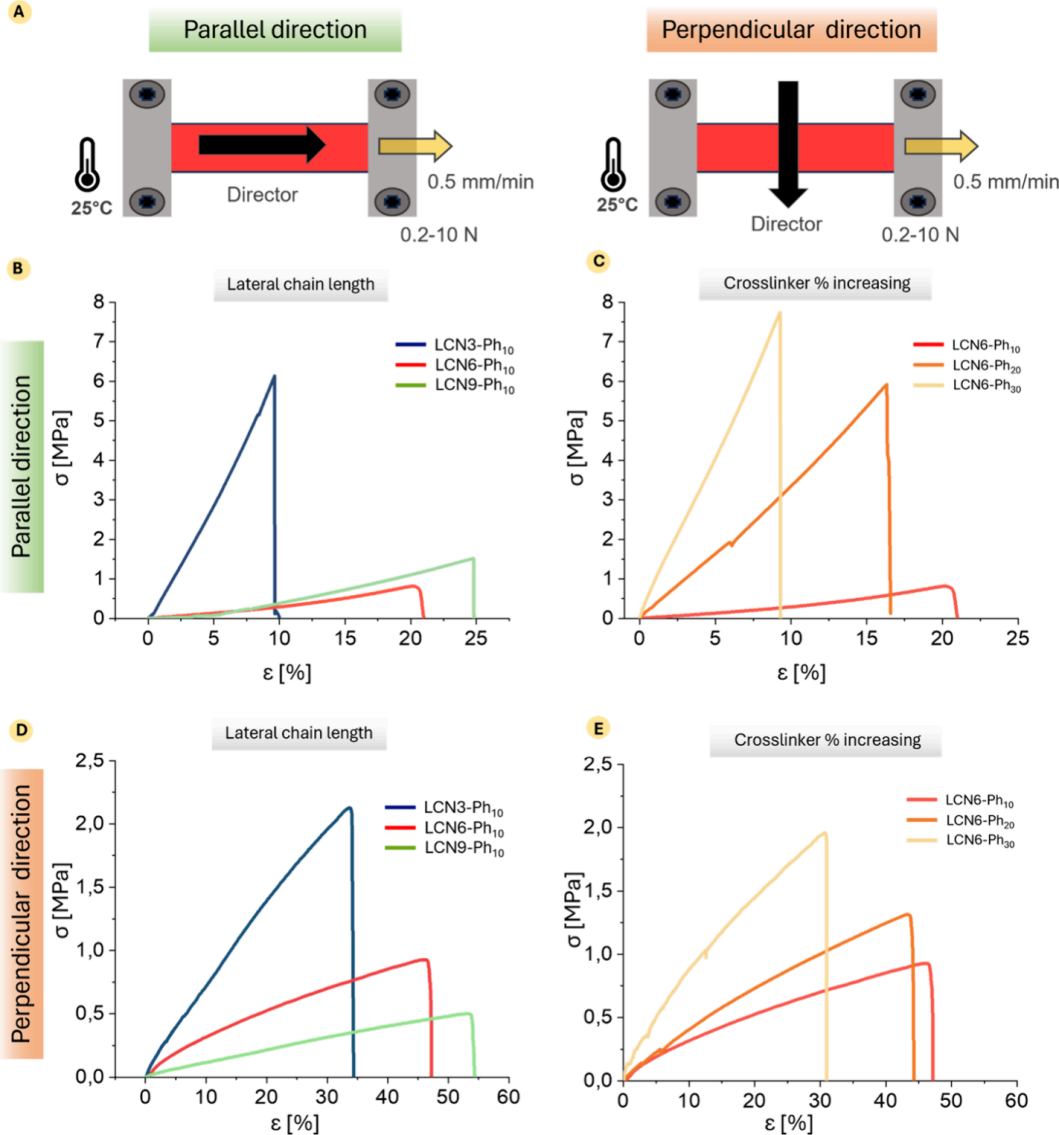
Tensile characterization of the LCNs. (A) Scheme of mechanical analysis setup. For each composition, tensile tests were performed in different directions with respect to the molecular alignment. (B–E) Representative stress–strain curves of the two series of materials analyzed: LCNs containing cross-linker with different chain lengths (on the left) and LCNs with different cross-linker content (on the right), in the parallel (B and C) and perpendicular (D and E) direction with respect to the molecular alignment. The curves reported are the average of 5 tests performed on different strips (taken from 5 different thin films).

A comparison of representative stress–strain curves for all films tested is shown in Figure 3. For each sample, the main mechanical parameters, namely Young’s modulus (*E*), stress at break (σ*), and strain at break (ε*), were extracted from the stress–strain curves. Data dispersion is shown in Figures S18 and S19 for tests performed in parallel and perpendicular directions, respectively, while the resulting average values and standard deviations are summarized in Table S2.

These data clearly show the role of the lateral chain length and cross-linker content in the material mechanical properties. Before discussing it more in detail, we highlight that, despite an inherent variability, most of the calculated mechanical properties can be considered statistically different (see Figures S18 and S19), thus providing robustness to the conclusions drawn from the observed trends. In particular, the extension of the lateral chain (Figure 3B,D) clearly affects the material stiffness, resulting in the stiffest LCN when the cross-linker presents the shorter lateral chain (LCN3-Ph_10_) while more flexible and deformable films can be obtained in the presence of longer side chains (e.g., using LCN6-Ph_10_ and LCN9-Ph_10_). Interestingly, this trend is more pronounced passing from 3 to 6 C atoms in the lateral chain (e.g., changing the cross-linker from CL3-Ph to CL6-Ph) than changing the lateral chain length from 6 to 9 C atoms (e.g., comparing materials containing LCN6-Ph_10_ or LCN9-Ph_10_), suggesting a sort of “saturation” effect. This behavior can be actually rationalized by considering that beyond a certain chain length, the added flexibility from additional methylene units no longer translates into meaningful changes in molecular mobility or packing disruption. In other words, once a threshold of conformational freedom is reached (around 6 carbons), further extension of the spacer does not significantly alter the polymer network’s ability to reorganize under strain. The same qualitative trend applies both in the parallel (Figure 3B) and perpendicular (Figure 3D) direction, although in the latter case, the property changes are more gradual. To be more quantitative, let us consider Young’s modulus, which experiences a marked reduction from ∼58 to ∼1 MPa in the parallel direction and from ∼10 to ∼4 MPa in the perpendicular direction (Table S2) when switching from LCN3-Ph_10_ to LCN6-Ph_10_, while it further reduces to only ∼0.8 to and ∼1.4 MPa for LCN9-Ph_10_ in the two test directions, respectively (note that differences in *E* values between LCN6-Ph_10_ or LCN-9 Ph_10_ for parallel direction are not even statistically significant, see Figure S18). Overall, we can conclude that the introduction of longer lateral chains can be exploited to improve elongation and flexibility of the material, at the expense of its stiffness. As expected,^36^ analogous considerations can be done when considering the effect of cross-linker concentration (Figure 3C–E). The increase of the cross-linker content from 10% to 30% allows to improve the material stiffness, leading to an increase of Young’s modulus and stress at break, combined with material embrittlement, as highlighted by a decrease of the strain at break (values are collected in Table S2). Again, although the overall trend is common to the two directions of analysis, the material properties in the parallel direction are the most affected ones by the amount of cross-linker. To sum up, the data in Figure 3 led to two main conclusions: (i) the mechanical behavior of LCNs is highly anisotropic, as already found in our previous work;^30^ (ii) the shorter the lateral chain length or the higher the cross-linker amount, the higher is the material stiffness and the lower is its deformability. However, mechanical characterization alone cannot drive the fine-tuning of the properties of the LCNs. For this reason, the study of the actuation under light stimulation was carried out as a complementary analysis, in order to deepen the understanding of the material properties not only in the passive state but also in the active one (under light stimulation to produce force).

### Light-Responsive Actuation of the LCN Films

When LCNs containing the azo-dye are exposed to an appropriate wavelength, they undergo contraction that varies based on the light intensity. This response can result from the *trans–cis* isomerization of the dye (photochemical effect) combined with the photothermal one.^13^

Indeed, the predominant actuation mechanism depends not only on the dye structure and content but also on the illumination conditions. In our case, DR1-acrylate undergoes *trans–cis* photoisomerization upon excitation in the blue region of the spectra (with a characteristic time scale of 0.5–1.4 ps) followed by a *cis–trans* relaxation in the millisecond range.^37,38^

Here, the photomechanical properties were evaluated in isometric conditions using a custom-made setup designed to measure the generation of tension and the kinetics of actuation under light stimulation. The system works mounting the LCN strips in between a force transducer at the top and a fixed support at the bottom (Figure 4A). The illumination was supplied by LEDs emitting at 470 nm, a wavelength carefully chosen to match the maximum absorption of the incorporated dye (with UV–vis spectra shown in Figure S20).^39,40^ This setup enables precise control over the material force production through light activation. Once the light stimulus is removed, the material spontaneously reverts to its original dimension, demonstrating that the process is completely reversible. Previous studies demonstrated a high fatigue resistance of the photocontractility, with materials working without showing any bleaching for at least 20 days with alternating irradiation and millions of cycles.^19^

**Figure 4:**
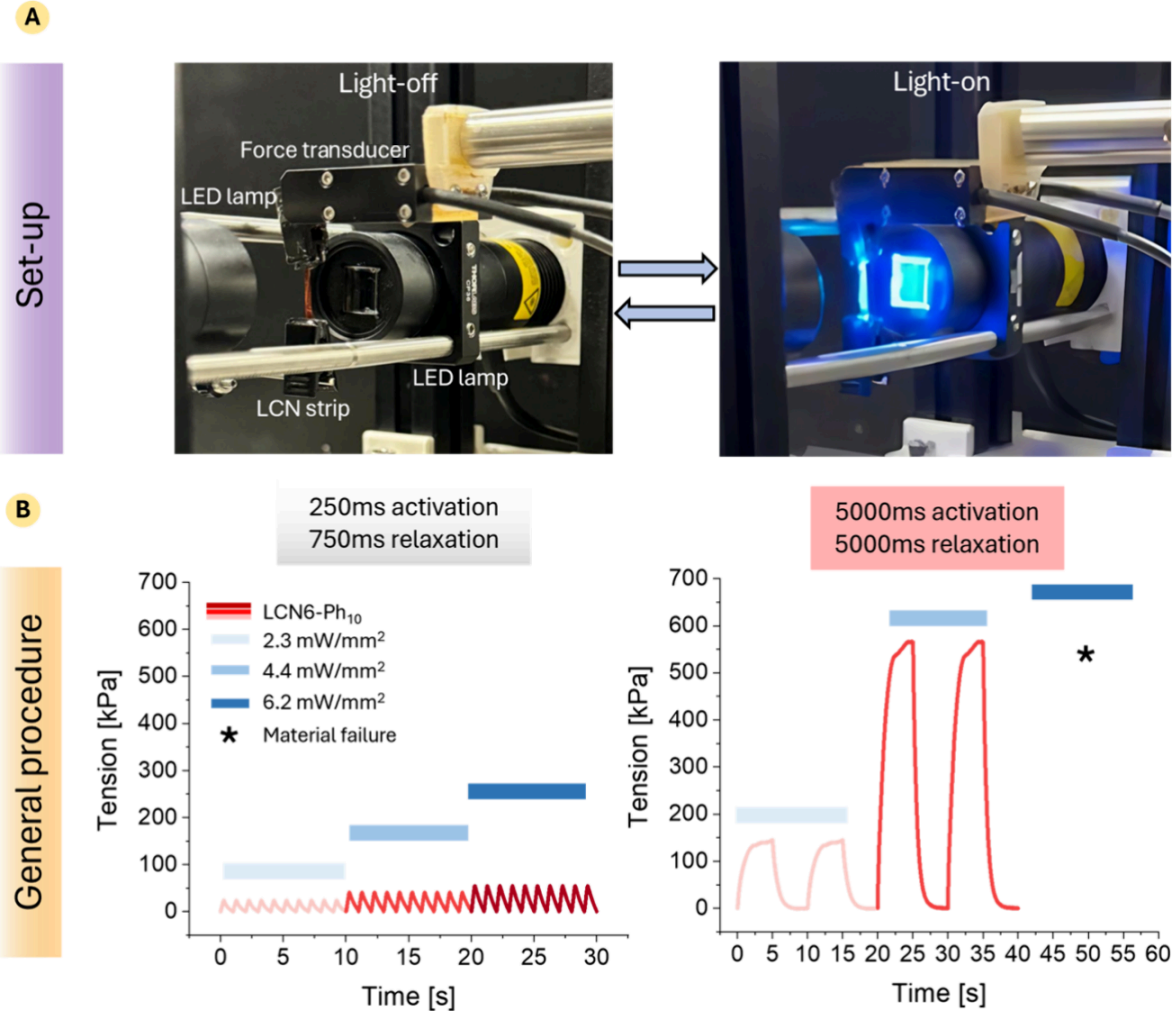
General description of the light actuation measurement. (A) Images of the setup used for the tension measurement. During the actuation, the sample was positioned in between the two LED lamps (blue light 470 nm); (B) trace of force development in multiple activation and relaxation cycles for LCN6-Ph_10_ during different irradiation times (250 and 5000 ms) and increasing the irradiation intensities (2.3, 4.4, and 6.2 mW/mm^2^).

To investigate the relationship between light dose and tension development, tests were conducted using two distinct light activation times: 250 ms (followed by 750 ms of relaxation with no light) and 5000 ms (followed by 5000 ms of relaxation). The use of these distinct activation times was motivated by their relevance in the fields of artificial muscles: the 250 ms activation aims to mimic the duration of a diastolic twitch in cardiac muscle, enabling a comparison with biological muscle function. However, this short activation time does not allow for the full development of the material’s contractile properties, such as the maximum developed force. Therefore, a longer activation period (5000 ms), which allows the system to reach a force plateau, was employed to enable a comprehensive characterization of the actuator’s mechanical performance.

The response of the materials was critically analyzed, revealing that the developed tension exhibited distinct patterns depending on the duration of light exposure. Figure 4B shows the force traces for LCN6-Ph_10_, taken here as an example, to explain the general protocol employed to test the light response. Each material has been subjected to multiple and consecutive activation–relaxation cycles, modifying both irradiation intensities (2.3, 4.4, and 6.2 mW/mm^2^) and activation times. The graphs demonstrate the ability to undergo multiple activation cycles without any loss of photomechanical performance, confirming the reliability in dynamic applications. The tension traces for the 250 ms activation reveal a rapid response to the applied stimulus which increases, increasing the light power. In contrast, the 5000 ms activation led to a marked increase in the tension generated up to a plateau value (maximum tension).

The influence of the spacer length on the actuation properties is shown in the graphs in Figure 5A (extracted maximum tension developed are reported in Tables S3 and S4). For all samples, both the maximum tension and the actuation rate increase when a higher irradiation intensity is applied. Considering the behavior at a fast illumination rate (250 ms of irradiation), the maximum tension produced (for all the intensity tested) first increased passing from a spacer with 3 carbons to the one with 6 carbons and then strongly decreased for a longer alkyl chain (e.g., 15 kPa for LCN3-Ph_10_ and 6 kPa for LCN9-Ph_10_ at 2.3 mW/mm^2^). On the other hand, for longer activation times, the behavior is totally different, with force that increases together with the spacer length. LCN9-Ph_10_ is the only formulation capable of supporting the higher irradiation intensity without mechanical failure and producing the maximum force recorded in all of the series tested here (tension developed 660 kPa at 6.2 mW/mm^2^). This phenomenon may be attributed to the increased flexibility afforded by longer spacers, allowing for greater structural deformation and enhanced tension generation during actuation.

**Figure 5:**
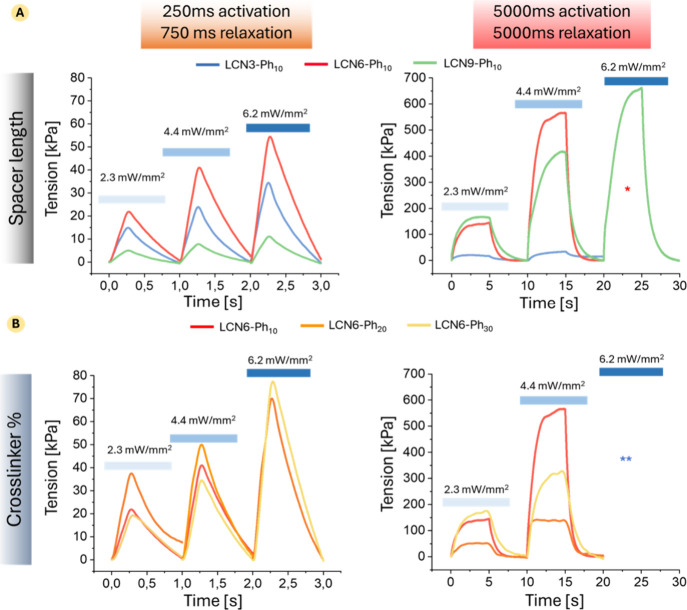
Traces of tension produced by different LCNs under light irradiation. (A) Effect of the spacer length on the tension development; (B) effect of the cross-linker content on the tension development. Data reported are representative traces obtained by irradiation of the material with three different irradiation intensities: 2.3, 4.4, and 6.2 mW/mm^2^ (light, middle, and dark blue bars, respectively) and two different activation/relaxation times 250/750 (left panel) and 5000/5000 ms (right panel). *LCN3-Ph_10_ and LCN6-Ph_10_ undergo mechanical failure at the highest irradiation intensity (6.2 mW/mm^2^). **All of the samples encounter a mechanical failure at the highest irradiation intensity (6.2 mW/mm^2^).

To assess the kinetic behavior of the materials, the activation and relaxation rates were extrapolated from the tension/time curves. A linear fit was applied in the initial linear region (first 100 ms of activation or relaxation) to determine the activation and relaxation rates (Figure S21) with values reported in Tables S5 and S6. Indeed, the speed increases in all samples using high irradiation intensity and, for short irradiation times, the speed is maximum for LCN6-Ph_10_ and minimum for LCN9-Ph_10_ (in line with the maximum force produced).

Furthermore, Figure 5B and Tables S7 and S8 provide insight into how varying the percentage of cross-linker influences the actuation performance. The results show significant differences in the maximum tensions achieved as a function of the cross-linker content at 250 ms activation time. For example, LCN6-Ph_10_ achieved a maximum average tension of 22 kPa at 2.3 mW/mm^2^, which increased to 40 kPa at 4.4 mW/mm^2^. At the same time, LCN6-Ph_20_ showed a higher performance with a maximum tension of 36 kPa at 2.3 mW/mm^2^, reaching 50 kPa at 4.4 mW/mm^2^. In this case, all the 3 samples can produce tension in the same value range without a drastic drop at a certain cross-linking degree. On the other hand, looking at traces with longer irradiation time, it is clear that the increase in the cross-linker amount leads to a sharp decrease in the actuation performance, with LCN6-Ph_10_ greatly exceeding the other formulations, demonstrating how the increase of cross-linker is not a good strategy to enhance the force production in this condition. Also in this series, none of the tested materials can withstand the higher light intensity tested (6.2 mW/mm^2^) for long activation times.

Looking at the activation rate at short illumination time, LCN6-Ph_20_ displayed better kinetics, beginning with 0.16 kPa/ms at 2.3 mW/mm^2^ and reaching 0.40 kPa/ms at 6.2 mW/mm^2^. A further increase of cross-linker molar content (30% mol/mol) or the extension of the lateral chain (LCN9-Ph_10_) showed a general decrease in the kinetic performance for short irradiation times.

Combining all of the results, LCN6-Ph_20_ achieves the better balance of mechanical strength and actuation performance. Under 250 ms light activation at 4.4 mW/mm^2^, it generated a tension of 50 kPa, outperforming LCN6-Ph_10_ (40 kPa) and LCN9-Ph_10_ (8 kPa).

Last but not least, the actuation mechanism also deserves to be considered. Indeed, the temperature of the material surface during light activation was observed by an infrared thermocamera. The results are presented in Figure S22 of the Supporting Information for a representative sample (LCN6-Ph_30_), both during short (250 ms) and long (5000 ms) light exposure cycles, and reported in Table S9 of the Supporting Information for all samples (250 ms activation). At short activation times, a maximum increase of 5 °C was observed, while for longer exposure, the material reaches temperatures up to 51 °C (increasing by about 26 °C). Therefore, even if in the first condition we could not exclude a significant effect of the photochemical isomerization (due to the low temperature increase in each cycle in correspondence with the force generation), for longer irradiation time, the deformation is primarily due to a photothermal effect. This observation is also consistent with previous reports on push–pull azobenzene-functionalized LCNs.^41^ To confirm the predominance of the photothermal effect, we also compared the force development trace with the real-time temperature curve (Figure S22), revealing how the mechanical response closely follows the temperature increase, without the typical lag of the photochemical strain. On the other hand, considering the physiological relevant temperature windows, the ability of our system to reach functional actuation without extreme thermal spikes (at high actuation rate, see Table S9, for maximum temperature reached) still suggests its potential suitability for biointegrated applications.^42^

## Conclusions

This study extends the remarkable capabilities of LCNs as advanced materials able to undergo fast and reversible light-induced shape-changes. By precisely modifying the molecular structure of the cross-linker, through lateral chain length modification and its content in the material formulation, we achieved a significant modulation of the mechanical and actuation properties. About the passive properties of the material (without stimulation), a higher cross-linker content or a shorter lateral chain allows enhancement of the material stiffness, showing an increase of both the Young modulus and the stress at break. More complex is the picture highlighted during the actuation test under light irradiation. All the materials can respond to short periods of light irradiation (250 ms) with a maximum tension up to 78 kPa (for LCN6-Ph_30_). The increase in the cross-linker chain length is detrimental to the sample LCN9-Ph_10_ that presents a poor force development, while increasing the cross-linker amount leads to a less marked modulation of the fast activation.

A different result is obtained for longer activation time, where the maximum tension of 660 kPa can be obtained with the cross-linker having the longest chain length. Indeed, the best combination of cross-linker structure and its amount needs to be carefully designed for each specific application, e.g., evaluating if we need to maximize the force or to improve the kinetics. We therefore believe that future research, focused on expanding the range of cross-linker modifications, will be highly desirable. Indeed, by leveraging light as both an actuation stimulus and a manufacturing tool, these materials can pave the path for ground-breaking innovations in light-responsive technologies spanning actuators for microrobotics to biomedical devices.

## Supplementary Material



## Notes

Additional experimental details, materials, methods, including DSC and POM images, and tables on mechanical and light actuation of LCNs, photothermal imaging under light-irradiation (PDF)

## ABBREVIATIONS

LC: liquid crystal
LCN: liquid crystalline network
Cr: crystalline phase
N: nematic phase
I: isotropic phase
RM257: 1,4-bis-[4-(3-acryloyloxypropyloxy)­benzoyloxy]-2-methylbenzene
RM82: 2-methyl-1,4-phenylene bis­(4-((6-(acryloyloxy)­hexyl)­oxy)­benzoate)
C6BP: 4-methoxybenzoic acid 4-(6-acryloyloxyhexyloxy) phenyl ester
DR1-acrylate: Disperse Red 1 acrylate
LED: light-emitting diode
DSC: differential scanning calorimetry
POM: polarized optical microscopy
ROI: region of interest
