# Supplementary material for: What Is the Right Chain Length? Liquid Crystalline Network Tuning by Molecular Design
Source: Macromolecules. 2025 Sep 11;58(18):9672–81. doi: 10.1021/acs.macromol.5c01011 (PMC12462244; doi:10.1021/acs.macromol.5c01011)
Supplement: Supplementary file 1 [file ma5c01011_si_001.pdf]

What is the right chain length? Liquid Crystalline Network tuning by molecular design

Simone Donato, Rachele Bini, Giovanni Simonetti, Neri Fuochi, Martina Salzano de Luna, Camille Chatard, Pierre-Louis Brient, Diederik S. Wiersma, Daniele Martella,* Camilla Parmeggiani*

Synthesis of LC crosslinkers

General methods. All reagents and solvents were commercially available and used as received. All reactions were carried out under magnetic stirring and monitored by ^{1}H-NMR. NMR spectra were recorded with a Bruker 300 MHz at 25°C. The chemical shift values (δ) are reported in ppm and relative to the TMS signal (δ= 0.00 ppm); the notations s, d, t, p, m indicate respectively: singlet, doublet, triplet, quintuplet, multiplet.

*Synthesis of Ethyl 4-(3-hydroxypropoxy)benzoate (**1**).* Ethyl 4-hydroxybenzoate (35.0 g, 0.21 mol) was dissolved in acetone (1.6 L) in a 2 L flask. Then, 3-bromo-1-propanol (33.7 g, 0.24 mol) and potassium carbonate (72.8 g, 0.53 mol) were added and the reaction was left at reflux for 18 h. The mixture was cooled to room temperature and filtrated. The filtrate was concentrated by evaporation under reduced pressure, then precipitated twice in hexane. After filtration and drying, the product was obtained as a white powder. Yield: 90% (42.5 g).

^{1}H-NMR (300 MHz, Methanol-d4) δ 8.06 – 7.84 (m, 2H), 7.13 – 6.87 (m, 2H), 4.34 (q, J = 7.1 Hz, 2H), 4.17 (t, J = 6.3 Hz, 2H), 3.76 (t, J = 6.2 Hz, 2H), 2.02 (p, J = 6.3 Hz, 2H), 1.38 (t, J = 7.1 Hz, 3H).

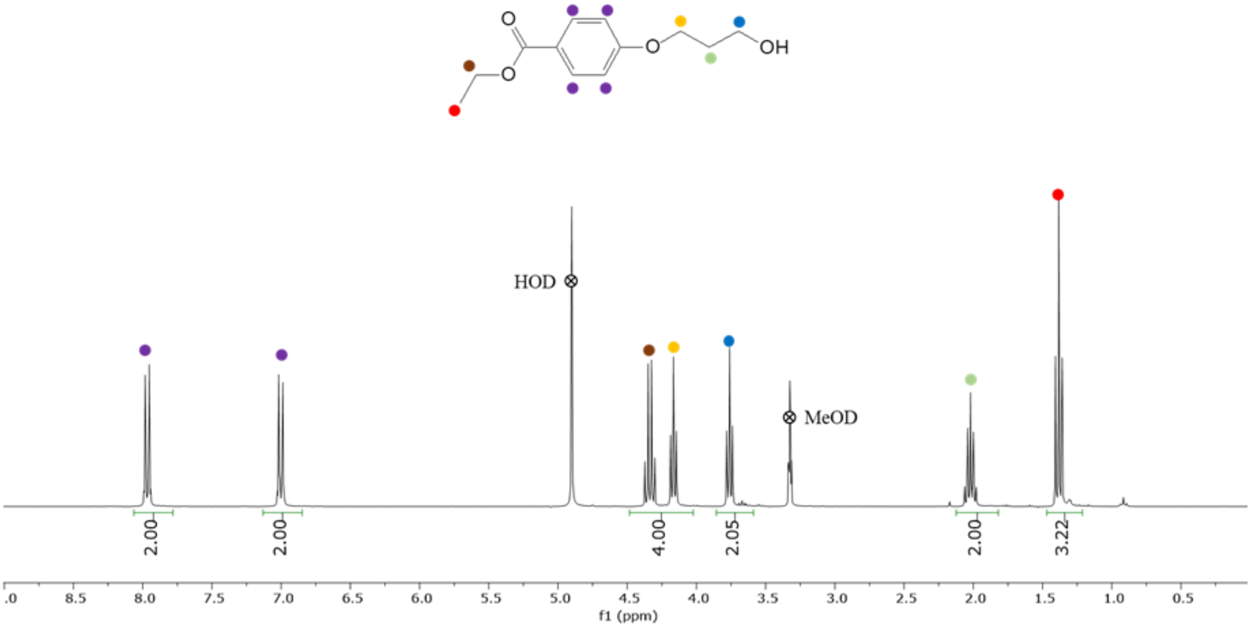

Figure S1: ^{1}H-NMR (300 MHz, Methanol-d4) of 1

*Synthesis of 4-(3-Hydroxypropoxy)benzoic acid (**2**).* In a 2 L flask, **1** (83.6 g, 0.37 mol) was dissolved in a mixture of ethanol (1 L) and deionized water (275 mL). Then, potassium hydroxide (41.8 g, 0.75 mol) was added and the reaction was left at reflux for 4 h. The mixture was concentrated by evaporation under reduced pressure, then acidified with a solution of HCl 0.5 M until pH=2-3 to give a white precipitate. After filtration and drying, the product **2** was obtained as a white powder. Yield: 97% (70.6 g).

^{1}H-NMR (300 MHz, DMSO-d6) δ 7.24 – 7.01 (m, 2H), 6.28 – 6.04 (m, 2H), 3.33 (t, J = 6.2 Hz, 2H), 2.93 (t, J = 6.2 Hz, 2H), 1.19 (p, J = 6.3 Hz, 2H).

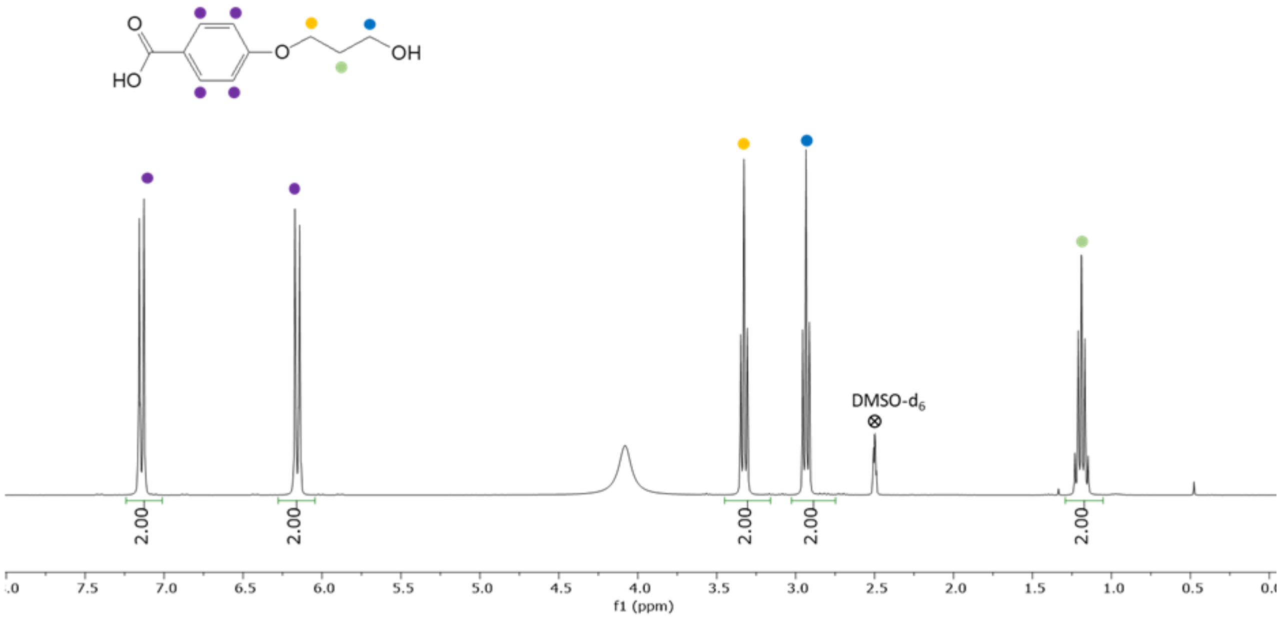

Figure S2: ^{1}H-NMR (300 MHz, DMSO-d6) of 2

*Synthesis of 4-(3-Acryloyloxypropyloxy)benzoic acid (**3**).* To an ice-cooled solution of **2** (59.7 g, 0.30 mol), N,N-dimethylaniline (44.2 g, 0.37 mol), 2,6-ditertbutyl-4-methylphenol (0.67 g, 3.0 mmol) in 1,4-dioxane (400 mL), acryloyl chloride (41.3 g, 0.46 mol) was added dropwise. The mixture was stirred at room temperature for 30 min, then warmed to 55 °C and stirred for 3 h. The solution was cooled to room temperature and poured into ice water. The white precipitate was filtered and washed several times with water. The product was then purified by recrystallization from ethanol to afford a white powder. Yield: 79% (60.0 g).

^{1}H-NMR (300 MHz, Chloroform-d) δ 8.18 – 7.99 (m, 2H), 7.08 – 6.88 (m, 2H), 6.44 (dd, J = 17.3, 1.5 Hz, 1H), 6.15 (dd, J = 17.3, 10.4 Hz, 1H), 5.86 (dd, J = 10.4, 1.5 Hz, 1H), 4.39 (t, J = 6.2 Hz, 2H), 4.16 (t, J = 6.1 Hz, 2H), 2.22 (p, J = 6.2 Hz, 2H).

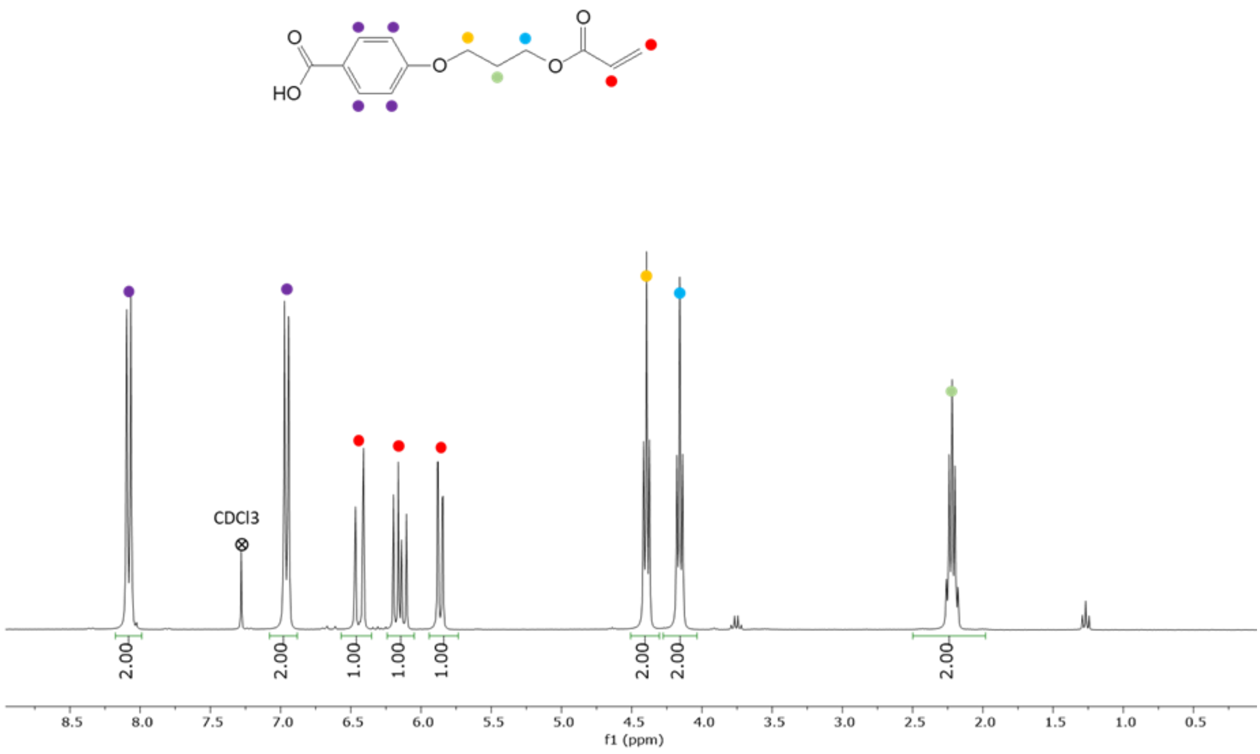

Figure S3: ^{1}H-NMR (300 MHz, Chloroform-d) of 3

Synthesis of CL3-Ph. In a 500 mL flask, **3** (16.3 g, 65 mmol), 2-phenylhydroquinone (5.5 g, 29.5 mmol), N-(3-dimetilaminopropil)-N'-etilcarbodiimide (EDC) hydrochloride (14.7 g, 76.8 mmol) and 4-(dimethylamino)pyridine (DMAP) (1.44 g, 11.8 mmol) were dissolved in anhydrous dichloromethane (115 mL). The reaction was stirred at room temperature for 16 h. After diluting the crude in dichloromethane, the organic phase was washed with a solution of HCl 0.5 M then deionized water. The combined organic layers were dried on anhydrous sodium sulfate and evaporated under reduced pressure. The crude was recrystallized from a mixture of isopropanol and hexane (1:1 vol), providing CL3-Ph as a beige powder. Yield: 62% (12.0 g).

^{1}H-NMR (300 MHz, Chloroform-d) δ 8.26 – 8.13 (m, 2H), 8.06 – 7.90 (m, 2H), 7.59 – 7.45 (m, 2H), 7.43 – 7.15 (m, 7H), 7.09 – 6.80 (m, 4H), 6.44 (dt, J = 17.2, 2.0 Hz, 2H), 6.15 (dd, J = 17.3, 10.4 Hz, 2H), 5.87 (dd, J = 10.4, 1.9 Hz, 2H), 4.40 (q, J = 6.4 Hz, 4H), 4.16 (dt, J = 12.9, 6.1 Hz, 4H), 2.22 (dt, J = 7.9, 6.0 Hz, 4H).

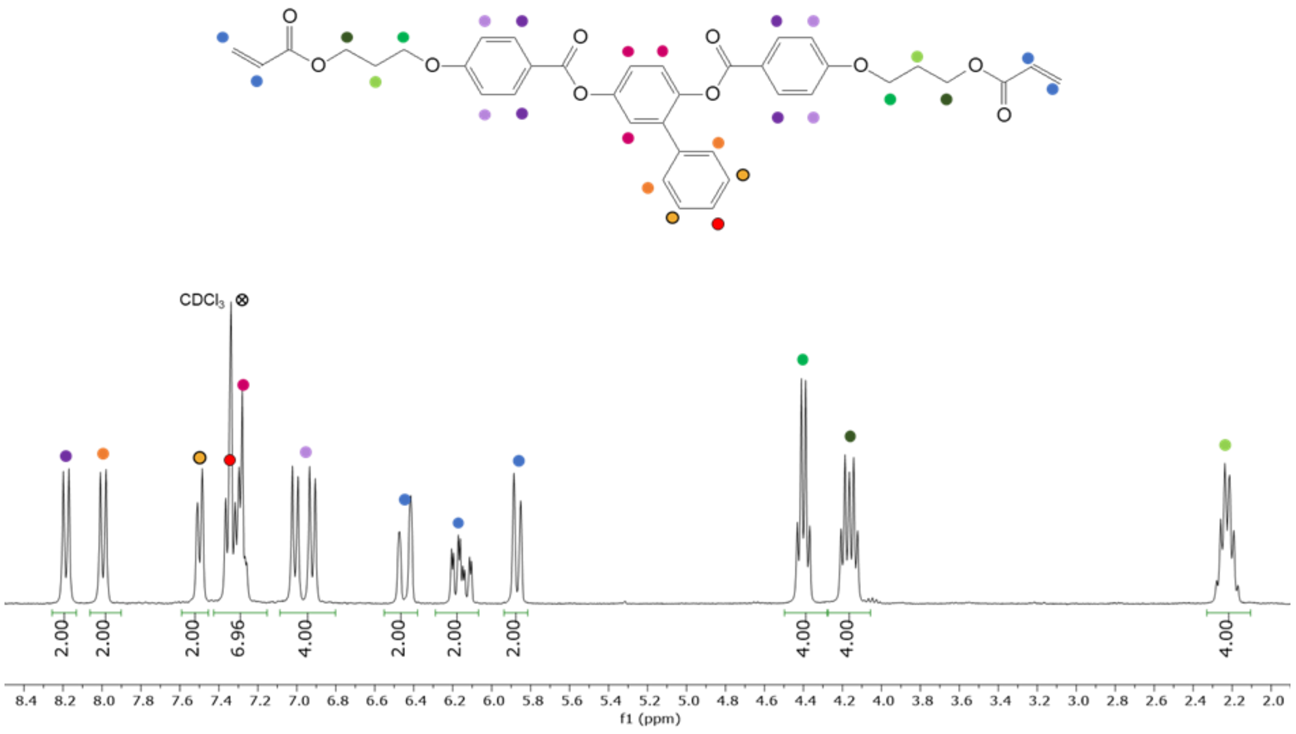

Figure S4: ^{1}H-NMR (300 MHz, Chloroform-d) of CL3-Ph

*Synthesis of Ethyl 4-(9-hydroxynonyloxy) benzoate (**4**)*. Ethyl 4-hydroxybenzoate (35.0 g, 0.21 mol) was dissolved in acetone (1.8 L) in a 2 L flask. Then, 9-bromo-1-nonanol (54.1 g, 0.24 mol) and potassium carbonate (72.8 g, 0.53 mol) were added and the reaction was left at reflux for 24 h. The mixture was cooled to room temperature and filtrated. The filtrate was concentrated by evaporation under reduced pressure, then precipitated twice in cold hexane. After filtration and drying, the product was obtained as a white powder. Yield: 92% (60.0 g).

^{1}H-NMR (300 MHz, Chloroform-d) δ 8.10 – 7.87 (m, 2H), 6.99 – 6.82 (m, 2H), 4.35 (q, J = 7.1 Hz, 2H), 4.00 (t, J = 6.5 Hz, 2H), 3.64 (td, J = 6.5, 5.1 Hz, 2H), 1.86 – 1.73 (m, 2H), 1.68 – 1.20 (m, 17H).

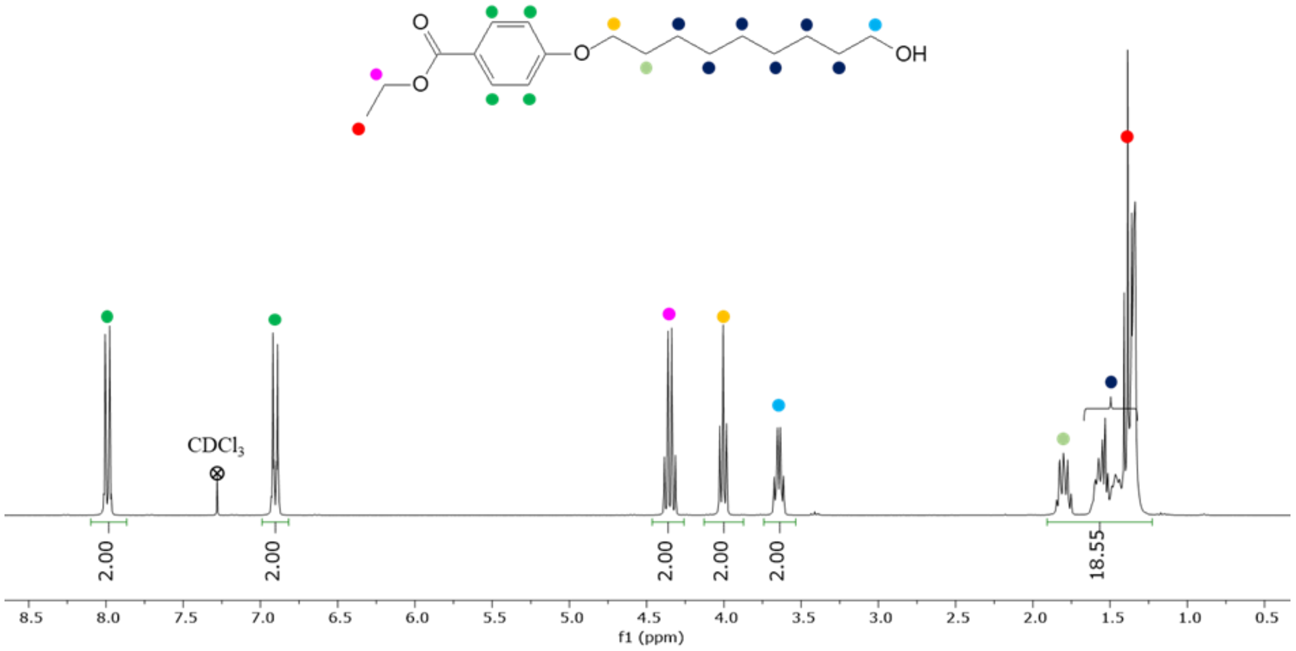

Figure S5: ^{1}H-NMR (300 MHz, Chloroform-d) of 4

*Synthesis of 4-(9-Hydroxynonyloxy)benzoic acid (**5**).* In a 2 L flask, **4** (57.0 g, 0.19 mol) was dissolved in a mixture of ethanol (700 mL) and deionized water (190 mL). Then, potassium hydroxide (20.7 g, 0.37 mol) was added and the reaction was left at reflux for 3 h. The mixture was concentrated by evaporation under reduced pressure, then acidified with a solution of HCl 0.5 M until pH=2-3 to give a white precipitate. After filtration and drying, the product **5** was obtained as a white powder. Yield: 95% (49.0 g).

^{1}H-NMR (300 MHz, DMSO-d6) δ 7.98 – 7.77 (m, 2H), 7.07 – 6.91 (m, 2H), 4.01 (t, J = 6.5 Hz, 2H), 3.37 (t, J = 6.4 Hz, 2H), 1.84 – 1.55 (m, 2H), 1.52 – 1.11 (m, 12H).

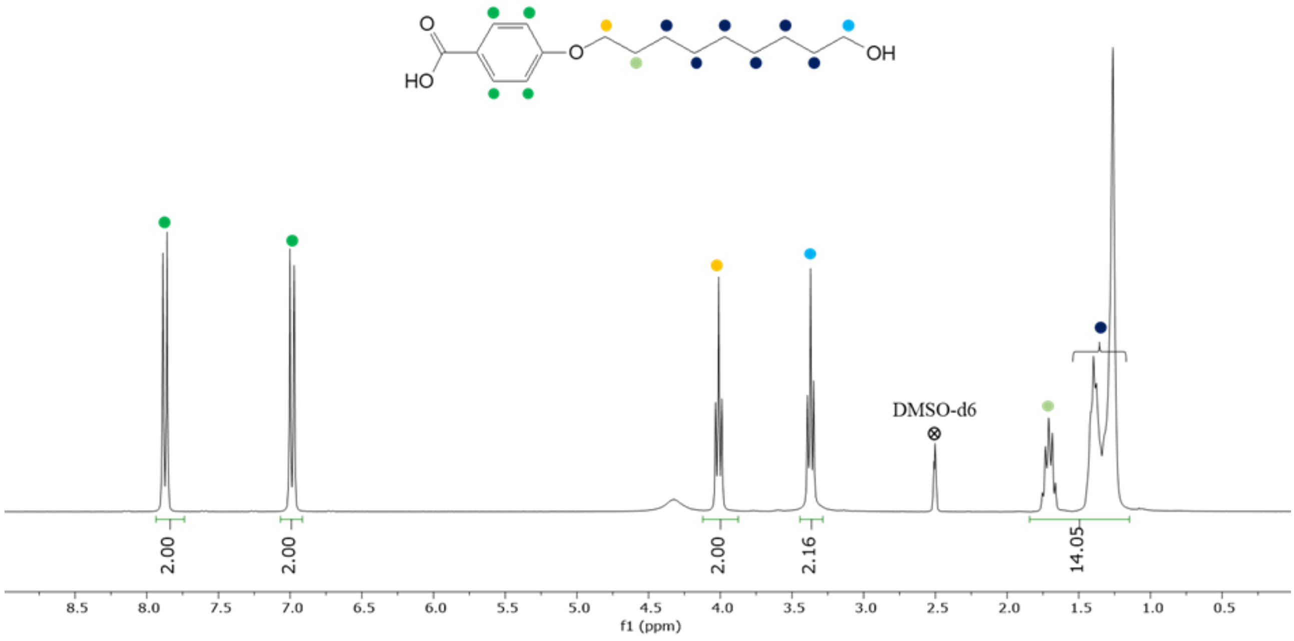

Figure S6: ^{1}H-NMR (300 MHz, DMSO-d6) of 5

*Synthesis of 4-(9-Acryloyloxynonyloxy)benzoic acid (**6**).* To an ice-cooled solution of **5** (90.0 g, 0.32 mol), N,N-dimethylaniline (46.7 g, 0.39 mol), 2,6-ditertbutyl-4-methylphenol (0.71 g, 3.2 mmol) in 1,4-dioxane (585 mL), acryloyl chloride (43.6 g, 0.48 mol) was added dropwise. The mixture was stirred at room temperature for 30 min, then warmed to 55 °C and stirred for 3 h. The solution was cooled to room temperature and poured into ice water. The white precipitate was filtered and washed several times with water. The product was then purified by recrystallization from ethanol to afford a white powder. Yield: 70% (75.0 g).

^{1}H-NMR (300 MHz, Chloroform-d) δ 8.13 – 7.93 (m, 2H), 7.03 – 6.85 (m, 2H), 6.40 (dd, J = 17.3, 1.6 Hz, 1H), 6.12 (dd, J = 17.3, 10.4 Hz, 1H), 5.82 (dd, J = 10.4, 1.6 Hz, 1H), 4.16 (t, J = 6.7 Hz, 2H), 4.02 (t, J = 6.5 Hz, 2H), 1.91 – 1.74 (m, 2H), 1.66 (q, J = 6.7 Hz, 2H), 1.36 (dt, J = 65.1, 7.0 Hz, 10H).

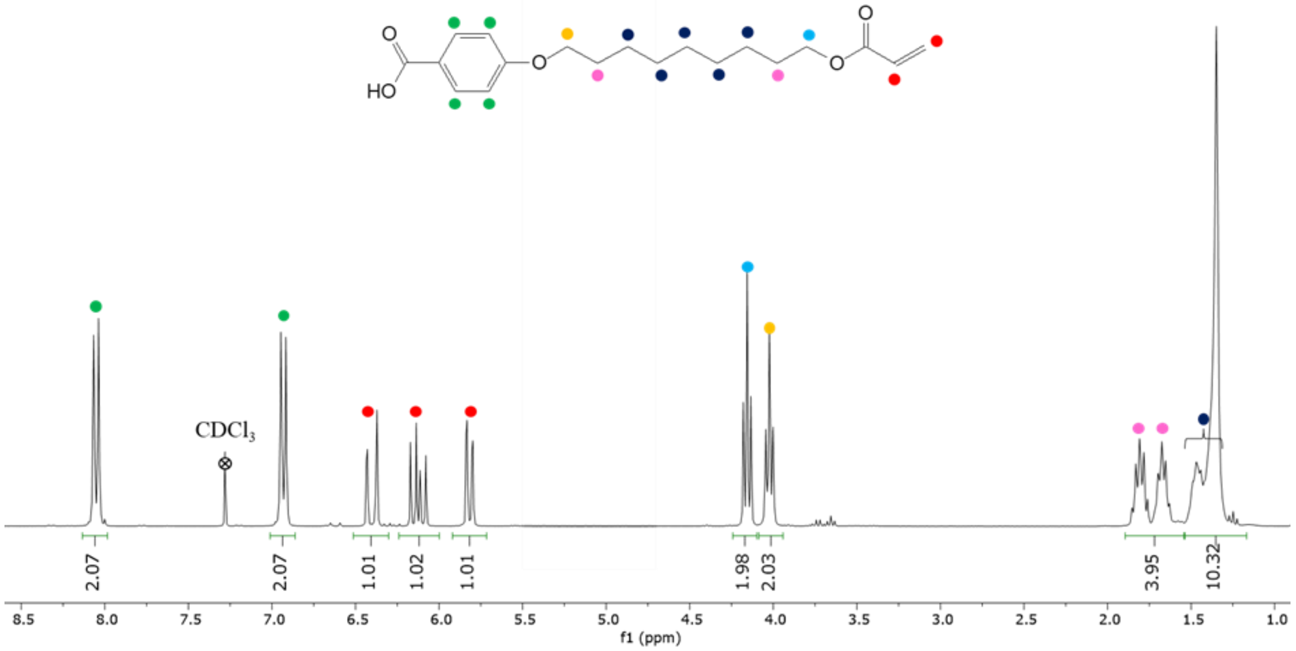

Figure S7: ^{1}H-NMR (300 MHz, Chloroform-d) of 6

Synthesis of CL9-Ph. In a 500 mL flask, **6** (19.8 g, 59.1 mmol), 2-phenylhydroquinone (5.0 g, 26.9 mmol), EDC hydrochloride (13.4 g, 69.8 mmol) and DMAP (1.31 g, 10.7 mmol) were dissolved in anhydrous dichloromethane (120 mL). The reaction was stirred at room temperature for 16 h. After diluting the crude in dichloromethane, the organic phase was washed with a solution of HCl 0.5 M then deionized water. The combined organic layers were dried on anhydrous sodium sulfate and evaporated under reduced pressure. The crude was recrystallized from a mixture of isopropanol and hexane (1:1 vol), providing CL9-Ph as a beige powder. Yield: 47% (10.3 g).

^{1}H-NMR (300 MHz, Chloroform-d) δ 8.29 – 8.09 (m, 2H), 8.09 – 7.86 (m, 2H), 7.64 – 7.43 (m, 2H), 7.41 – 7.19 (m, 6H), 7.10 – 6.82 (m, 4H), 6.42 (dd, J = 17.3, 1.6 Hz, 2H), 6.14 (dd, J = 17.3, 10.4 Hz, 2H), 5.83 (dd, J = 10.4, 1.6 Hz, 2H), 4.28 – 3.92 (m, 8H), 1.94 – 1.29 (m, 28H).

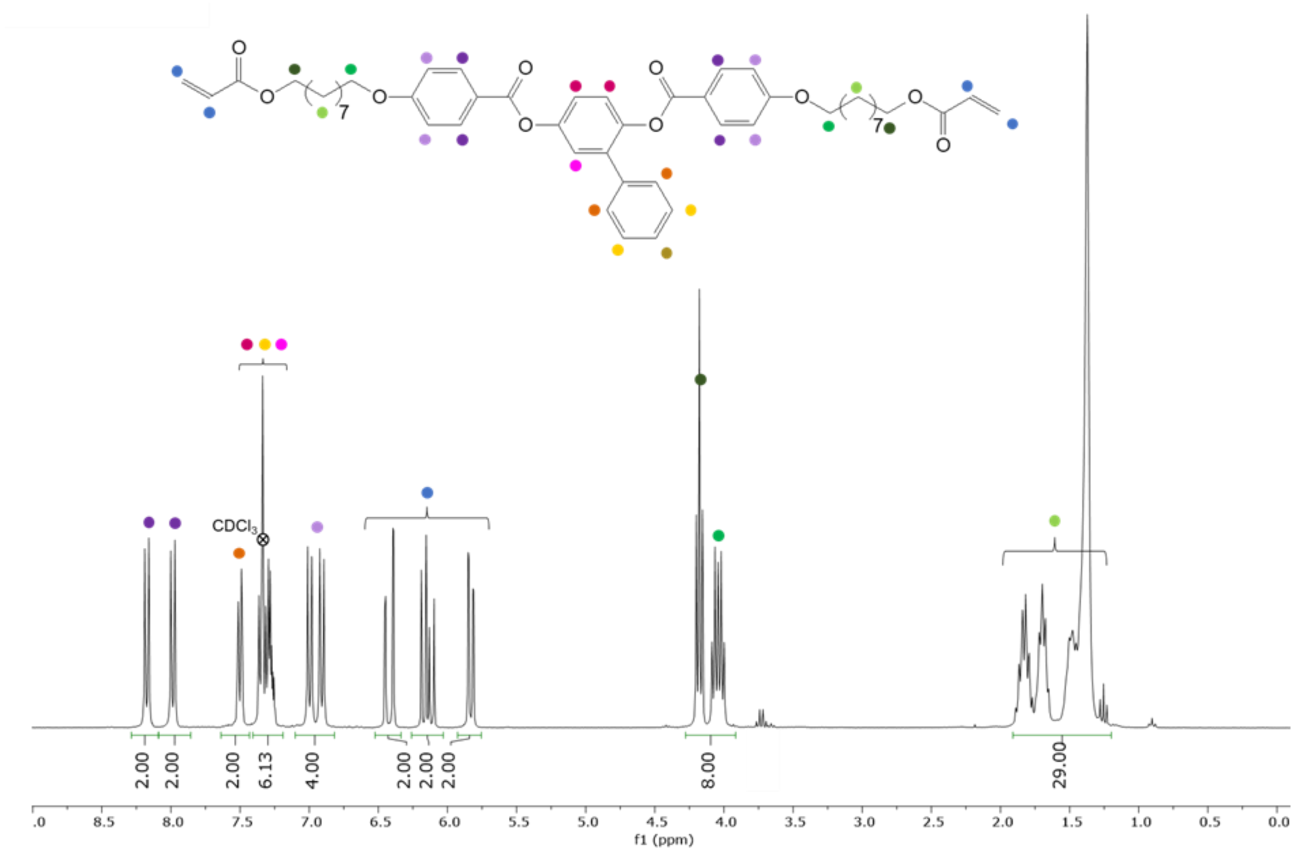

Figure S8: ^{1}H-NMR (300 MHz, Chloroform-d) of CL9-Ph

Mesomorphic properties of liquid crystalline crosslinkers

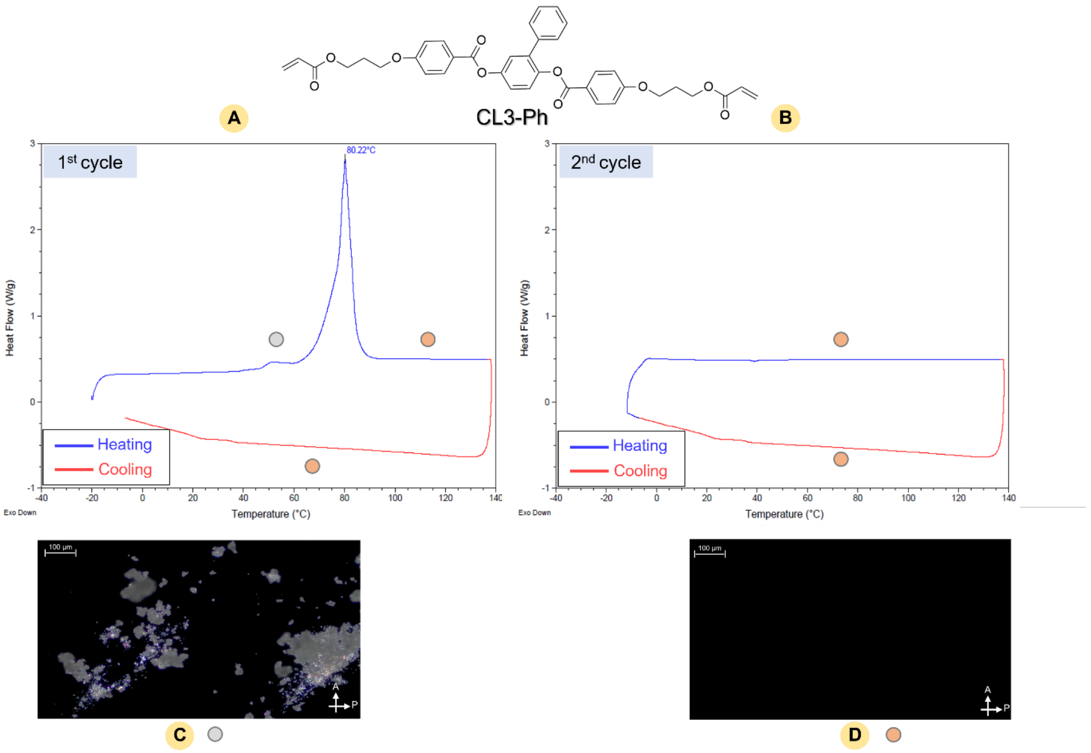

Figure S9: Mesomorphic properties of the CL3-Ph. A) DSC trace related to the first heating and cooling cycle; B) DSC trace related to the second heating and cooling cycle; C) POM image of the crystal phase at 35 °C on heating; D) POM image of the isotropic phase at 90 °C on cooling.

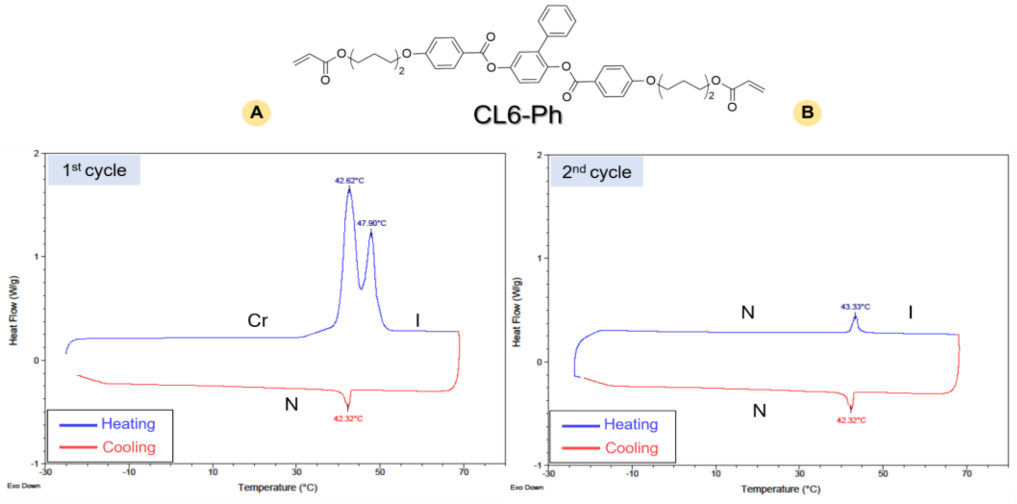

Figure S10: Mesomorphic properties of the CL6-Ph. A) DSC trace related to the first heating and cooling cycle; B) DSC trace related to the second heating and cooling cycle. Cr: Crystalline phase N: Nematic phase; I: Isotropic phase.

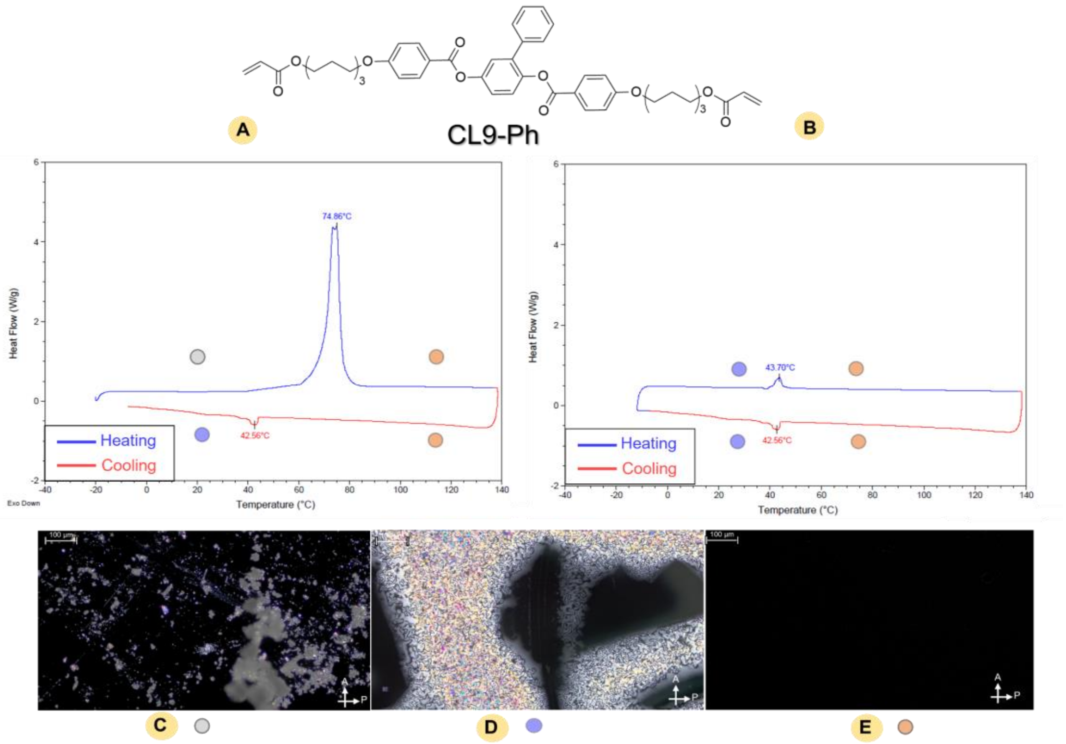

Figure S11: Mesomorphic properties of the CL9-Ph. A) DSC trace related to the first heating and cooling cycle; B) DSC trace related to the second heating and cooling cycle; C) POM image of the crystal phase at 40 °C on heating; D) POM image of the nematic phase at 30°C on cooling; E) POM image of the isotropic phase at 50°C on cooling.

Table S1: Composition of the monomeric mixtures. All quantities are indicated on %mol/mol of the entire mixture.

**Monomeric mixture**	C6BP [% mol/mol]	CL3-Ph [% mol/mol]	CL6-Ph [% mol/mol]	CL9-Ph [% mol/mol]	Irgacure 369 [%mol/mol]	DR1-A [% mol/mol]
LCN3-Ph10	88	10	-	-	1	1
LCN6-Ph10	88	-	10	-	1	1
LCN6-Ph20	78	-	20	-	1	1
LCN6-Ph30	68	-	30	-	1	1
LCN9-Ph10	88	-	-	10	1	1

Mesomorphic properties of liquid crystalline mixtures

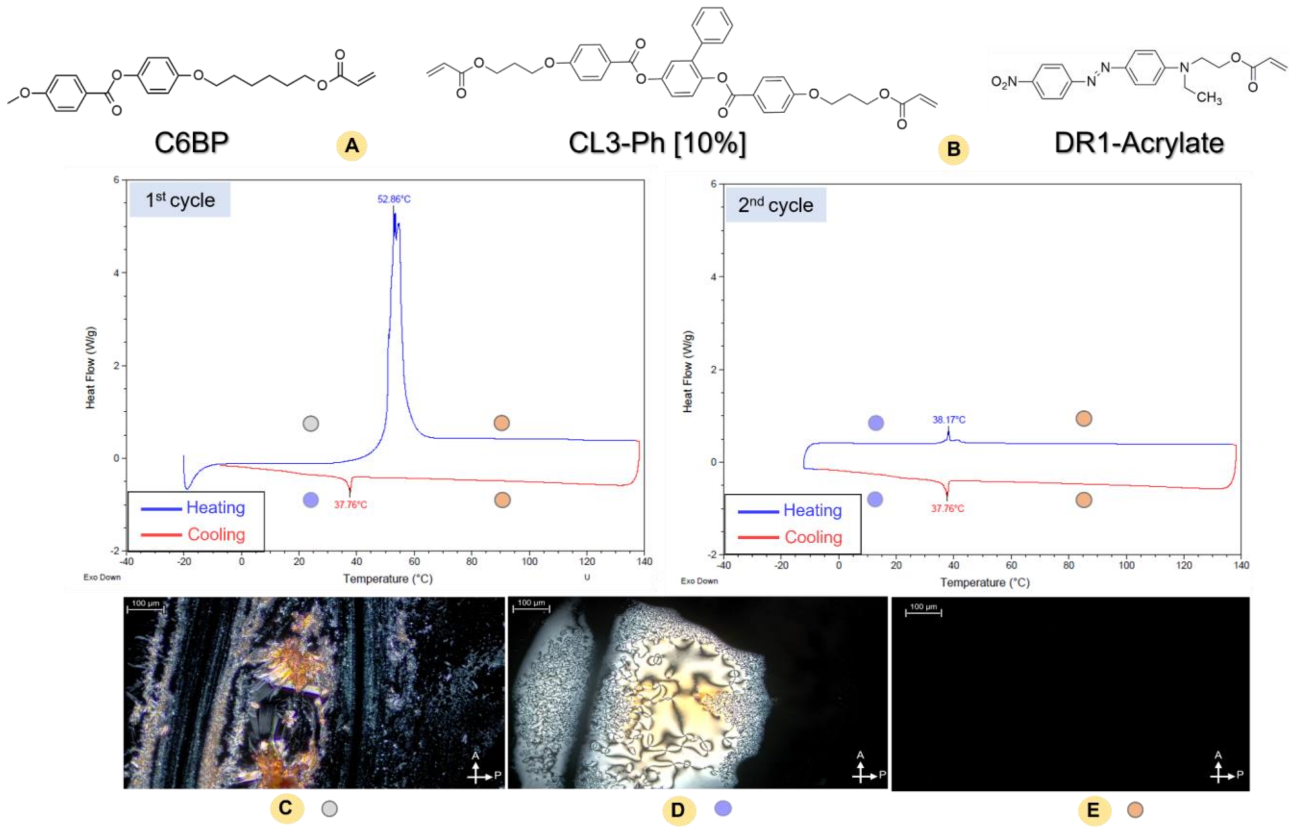

Figure S12: Mesomorphic properties of the mixture LCN3-Ph$_{10}$. A) DSC trace related to the first heating and cooling cycle; B) DSC trace related to the second heating and cooling cycle; C) POM image of the crystal phase at 40 °C on heating; D) POM image of the nematic phase at 35 °C on cooling; E) POM image of the isotropic phase at 50 °C on cooling.

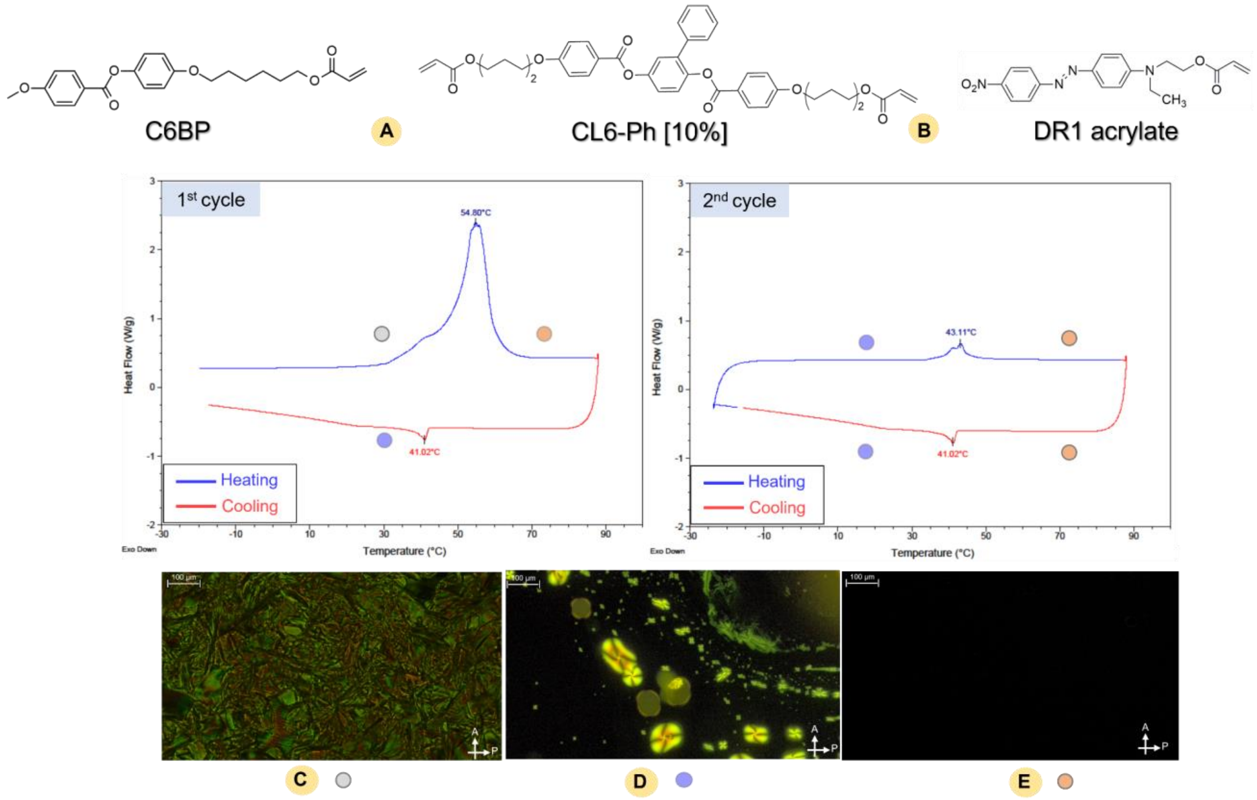

Figure S13: Mesomorphic properties of the mixture LCN6-Ph$_{10}$. A) DSC trace related to the first heating and cooling cycle; B) DSC trace related to the second heating and cooling cycle; C) POM image of the crystal phase at 45 °C on heating; D) POM image of the nematic phase at 40 °C on cooling; E) POM image of the isotropic phase at 50 °C on cooling.

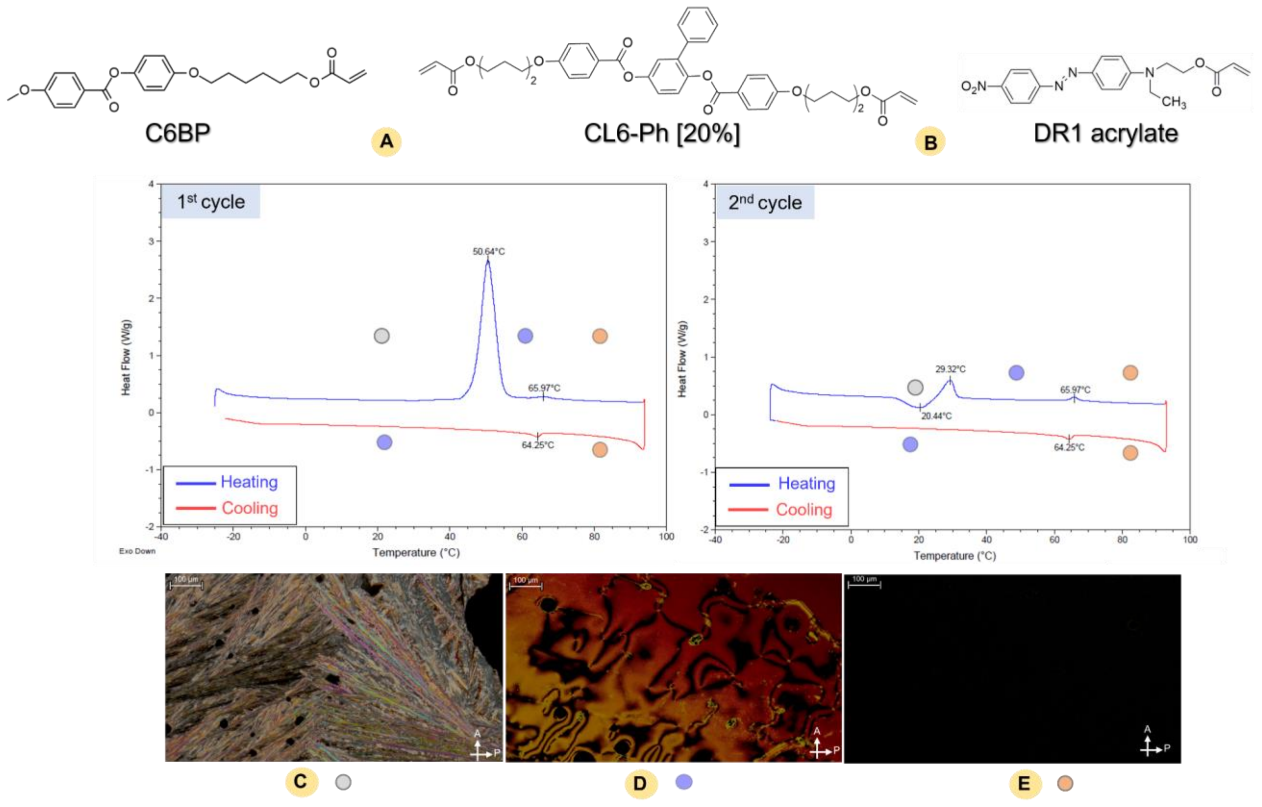

Figure S14: Mesomorphic properties of the mixture LCN6-Ph20. A) DSC trace related to the first heating and cooling cycle; B) DSC trace related to the second heating and cooling cycle; C) POM image of the crystal phase at 40°C on heating; D) POM image of the nematic phase at 50 °C on cooling; E) POM image of the isotropic phase at 70 °C on cooling. It was observed a cold crystallization on the second cycle of heating at 21°C.

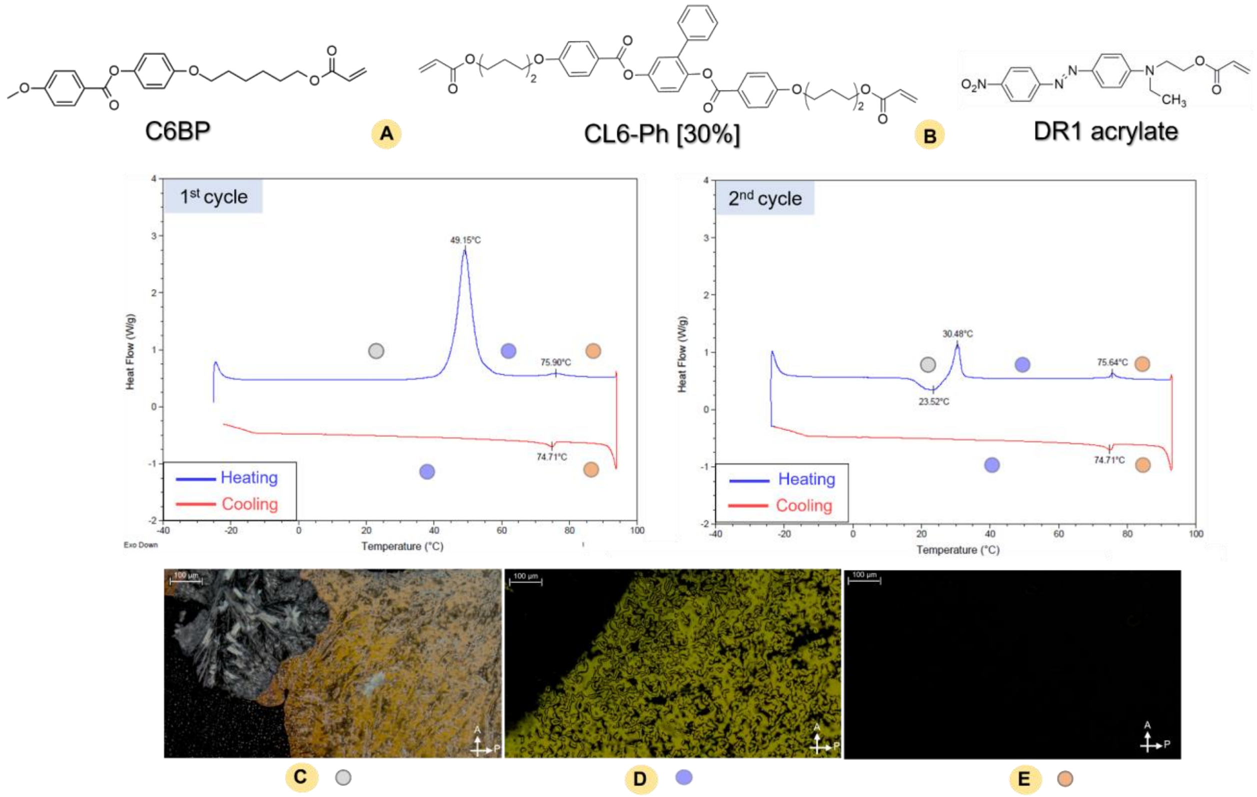

Figure S15: Mesomorphic properties of the LCN6-Ph30. A) DSC trace related to the first heating and cooling cycle; B) DSC trace related to the second heating and cooling cycle; C) POM image of the crystal phase at 45 °C on heating; D) POM image of the nematic phase at 50 °C on cooling; E) POM image of the isotropic phase at 75 °C on cooling. It was observed a cold crystallization on the second cycle of heating at 24°C.

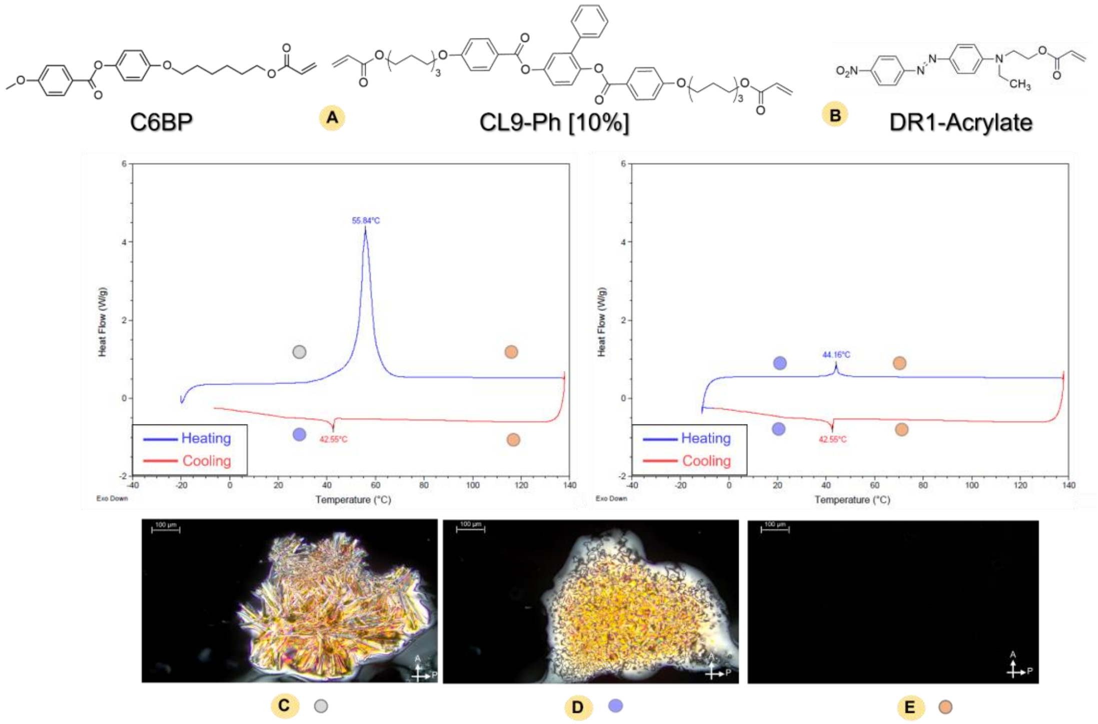

Figure S16: Mesomorphic properties of the LCN9-Ph10. A) DSC trace related to the first heating and cooling cycle; B) DSC trace related to the second heating and cooling cycle; C) POM image of the crystal phase at 40 °C on heating; D) POM image of the nematic phase at 35 °C on cooling; E) POM image of the isotropic phase at 60 °C on cooling.

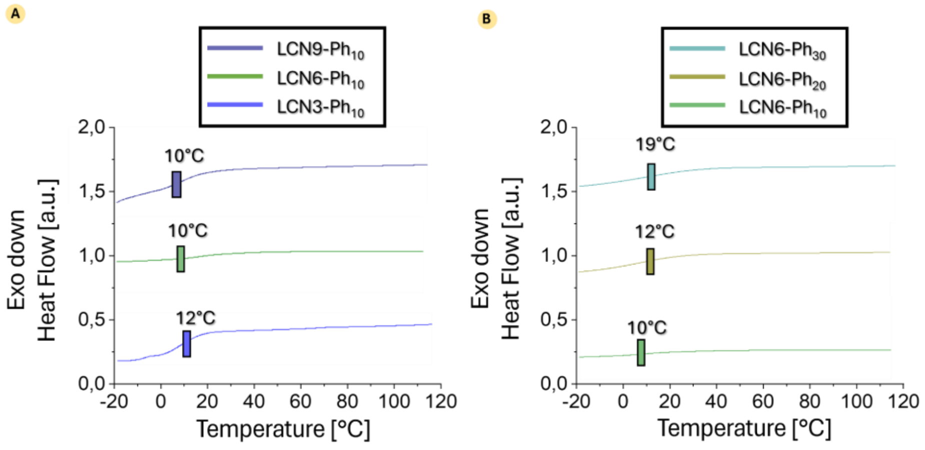

Figure S17: DSC traces of the LCN films. The graphs show the second heating cycle.

Mechanical properties of LCNs

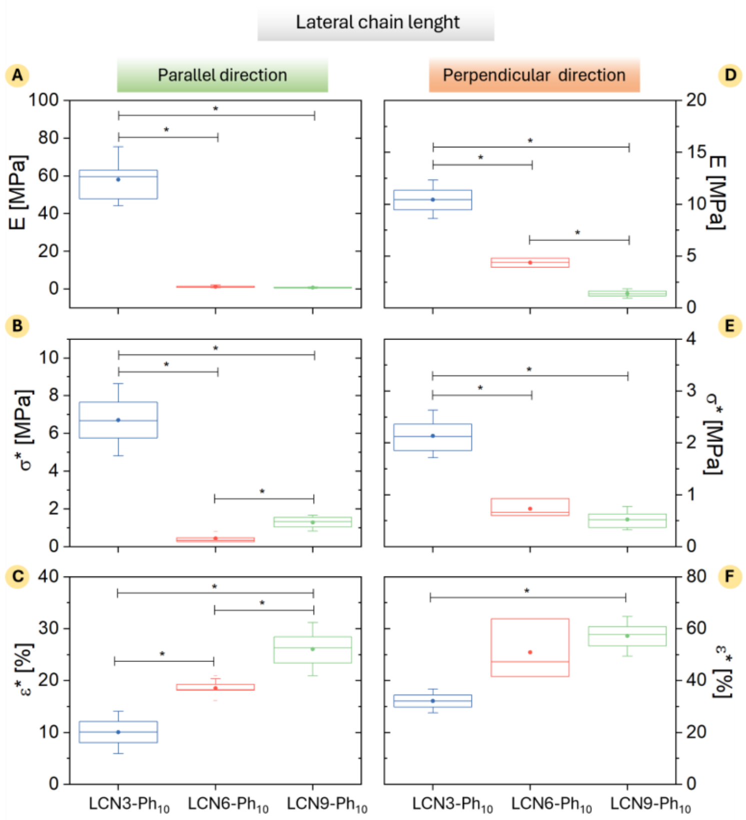

Figure S18: Box plots showing the variability of a,d) Young's modulus, b,e) stress at break, and c-f) elongation at break data as a function of the lateral chain length. Data reported for tensile tests carried out both in a-c) parallel and d-f) perpendicular direction. Data pairs marked with an asterisk are statistically different (t-test, $p<0.05$).

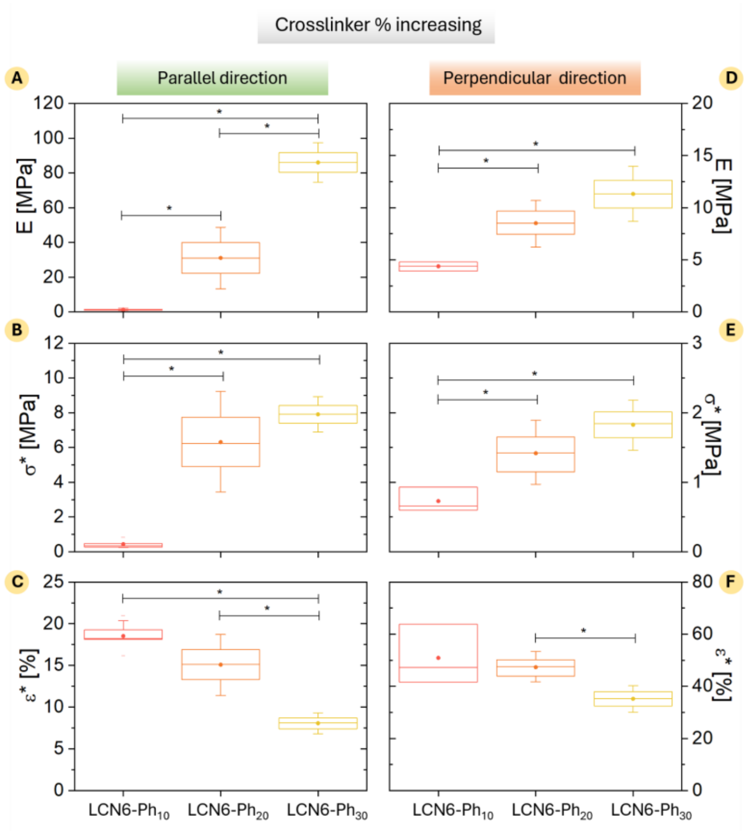

Figure S19: Box plots showing the variability of a,d) Young's modulus, b,e) stress at break, and c-f) elongation at break data as a function of the amount of crosslinker. Data reported for tensile tests carried out both in a-c) parallel and d-f) perpendicular direction. Data pairs marked with an asterisk are statistically different (t-test, $p<0.05$).

Table S2: Mechanical parameters of LCNs analyzed in two different directions (parallel and perpendicular with respect to the nematic director). Average and standard deviation of stress at break σ* (MPa), strain at break ε* (%) and Young modulus E (MPa).

	Parallel direction			Perpendicular direction		
**LCN**	σ* [MPa]	ε* [%]	E [MPa]	σ* [MPa]	ε* [%]	E [MPa]
LCN3-Ph_{10}	6.7±1.5	10±3.2	58±12.5	2±0.4	32±3.6	10.4±1.5
LCN6-Ph_{10}	0.4±0.1	18±2	1±0.5	0.7±0.2	51±11	4±0.5
LCN6-Ph_{20}	6.3±2.3	15±3	31±14	1.4±0.4	47±5	8.5±1.8
LCN6-Ph_{30}	8±0.8	8±1	86±9	1.8±0.3	35±4	11.3±2.1
LCN9-Ph_{10}	1.3±0.4	26±4	0.8±0.3	0.5±0.2	57±6	1.4±0.4

Light-actuation characterization of Liquid Crystalline Networks

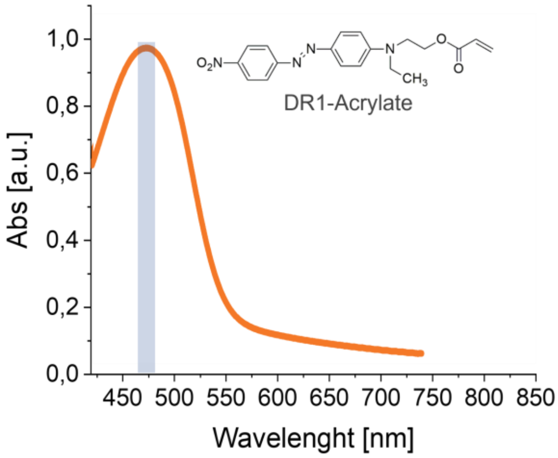

Figure S20: UV-Vis spectra of DR1-acrylate.

Table S3: Tension production for short irradiation time using different light intensities. T: average of the maximum tension calculated by dividing the measured maximum force for the illumination cross-section (0.03 mm thickness x 2 mm width) of the material; Values are the medium of 10 traces acquired for three different strips (each of them cut from a different sample). The light activation time is set to 250 ms (with 750 ms of relaxation).

**LCN | 250 ms**	T [kPa] 2.3 mW/mm^2	T[kPa] 4.4 mW/mm^2	T[kPa] 6.2 mW/mm^2
LCN3-Ph_{10}	15 ± 1	24 ± 0.6	34 ± 0.7
LCN6-Ph_{10}	22 ± 0.3	40 ± 0.8	54 ± 0.4
LCN6-Ph_{20}	36 ± 4	50 ± 0.1	70 ± 1.3
LCN6-Ph_{30}	19 ± 0.6	35 ± 2.7	78 ± 2
LCN9-Ph_{10}	6 ± 0.1	8 ± 0.1	12 ± 0.2

Table S4: Tension production for long irradiation using different irradiation intensities. T: Average of the maximum tension calculated by dividing the measured maximum force for the illumination cross-section (0.03 mm thickness x 2 mm width) of the material; Values are the medium of 10 traces acquired for three different strips (each of them cut from a different sample). The light activation time is set to 5000 ms (with 5000 ms of relaxation). * indicate the mechanical failure of the LCN.

**LCN | 5000 ms**	T [kPa] 2.3 mW/mm^2	T[kPa] 4.4 mW/mm^2	T[kPa] 6.2 mW/mm^2
LCN3-Ph_{10}	18 ± 8	35 ± 11	*
LCN6-Ph_{10}	146 ± 46	546 ± 79	*
LCN6-Ph_{20}	59 ± 4	144 ± 3	*
LCN6-Ph_{30}	185 ± 25	293 ± 21	*
LCN9-Ph_{10}	164 ± 22	415 ± 70	660 ± 100

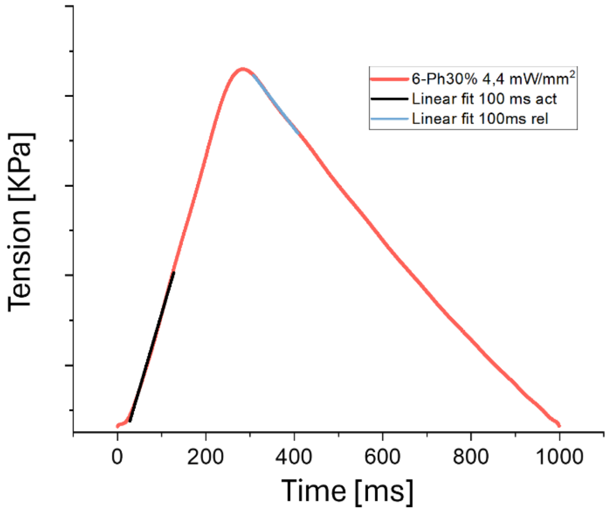

Figure S21: Example of the kinetic evaluation on light-actuation of LCN6-Ph_{30}. Linear fit curve for activation and relaxation (100ms) of the tension trace.

Table S5: Influence of lateral chain lengths on the actuation kinetic for short irradiation times. Va_{100}: Rate of activation of the material determined with a linear fit on the force development trace in the first 100 ms (after switching on the lamp); Vr_{100}: Rate of relaxation of the material determined with a linear fit on the force development trace in the first 100 ms (after switching off the lamp). Values are the medium of 10 traces acquired for three different strips (each of them cut from a different sample). In all tests, irradiation intensity was varied while the activation time was set to 250 ms (followed by 750 ms without irradiation to observe the material relaxation).

	Rate [kPa/ms]					
**LCN|250 ms**	Va_{100} 2.3 mW/mm^2	Va_{100} 4.4 mW/mm^2	Va_{100} 6.2 mW/mm^2	Vr_{100} 2.3 mW/mm^2	Vr_{100} 4.4 mW/mm^2	Vr_{100} 6.2 mW/mm^2
LCN3-Ph_{10}	0.07± 0.01	0.11± 0.003	0.16± 0.02	0.03 ± 0.004	0.06± 0.004	0.08 ± 0.001
LCN6-Ph_{10}	0.09± 0.001	0.18± 0.005	0.24± 0.002	0.03± 0.001	0.07± 0.003	0.08± 0.001
LCN9-Ph_{10}	0.03± 0.004	0.04 ± 0.006	0.06 ± 0.001	0.01 ± 0.006	0.02 ± 0.006	0.03 ± 0.001

Table S6: Influence of crosslinker content on the actuation kinetic for short irradiation times. Va_{100}: Rate of activation of the material determined with a linear fit on the force development trace in the first 100 ms (after switching on the lamp); Vr_{100}: Rate of relaxation of the material determined with a linear fit on the force development trace in the first 100 ms (after switching off the lamp). Values are the medium of 10 traces acquired for three different strips (each of them cut from a different sample). In all tests, irradiation intensity was varied while the activation time was set to 250 ms (followed by 750 ms without irradiation to observe the material relaxation).

	Rate [kPa/ms]					
**LCN|250 ms**	Va_{100} 2.3 mW/mm^2	Va_{100} 4.4 mW/mm^2	Va_{100} 6.2 mW/mm^2	Vr_{100} 2.3 mW/mm^2	Vr_{100} 4.4 mW/mm^2	Vr_{100} 6.2 mW/mm^2
LCN6-Ph_{10}	0.09± 0.001	0.18± 0.005	0.24± 0.002	0.03± 0.001	0.07± 0.003	0.08± 0.001
LCN6-Ph_{20}	0.16± 0.03	0.26 ± 0.003	0.4± 0.004	0.07 ± 0.007	0.1 ± 0.001	0.15 ± 0.003
LCN6-Ph_{30}	0.07 ± 0.002	0.14 ± 0.006	0.32 ± 0.008	0.03 ± 0.001	0.06± 0.001	0.12 ± 0.006

Table S7: Influence of lateral chain lengths on the actuation kinetic for long irradiation times. Va_{100}: Rate of activation of the material determined with a linear fit on the first 100 ms; Vr_{100}: Rate of relaxation of the material determined with a linear fit on the first 100 ms. The reported data are an average of 10 different curves for three different samples. Irradiation intensity was varied according to the tension development tests and the light-activation time was set to 5000 ms (5000 ms on relaxation). * indicate the mechanical failure of the LCN.

	Rate [kPa/ms]					
**LCN|5000 ms**	Va_{100} 2.3 mW/mm^2	Va_{100} 4.4 mW/mm^2	Va_{100} 6.2 mW/mm^2	Vr_{100} 2.3 mW/mm^2	Vr_{100} 4.4 mW/mm^2	Vr_{100} 6.2 mW/mm^2
LCN3-Ph_{10}	0.02± 0.001	0.03± 0.001	*	0.02± 0.001	0.03± 0.02	*
LCN6-Ph_{10}	0.17± 0.001	0.54± 0.06	*	0.2± 0.011	0.93± 0.01	*
LCN9-Ph_{10}	0.15± 0.001	0.30 ± 0.06	0.45 ± 0.20	0.14 ± 0.015	0.50 ± 0.04	0.91 ± 0.006

Table S8: Influence of crosslinker content on the actuation kinetic for long irradiation times. Va_{100}: Rate of activation of the material determined with a linear fit on the first 100 ms; Vr_{100}: Rate of relaxation of the material determined with a linear fit on the first 100 ms. The reported data are an average of 10 different curves for three different samples. Irradiation intensity was varied according to the tension development tests and the light-activation time was set to 5000 ms (5000 ms on relaxation). * indicate the mechanical failure of the LCN.

	Rate [kPa/ms]					
**LCN|5000 ms**	Va_{100} 2.3 mW/mm^2	Va_{100} 4.4 mW/mm^2	Va_{100} 6.2 mW/mm^2	Vr_{100} 2.3 mW/mm^2	Vr_{100} 4.4 mW/mm^2	Vr_{100} 6.2 mW/mm^2
LCN6-Ph_{10}	0.17± 0.001	0.54± 0.06	*	0.2± 0.011	0.93± 0.01	*
LCN6-Ph_{20}	0.06± 0.006	0.31 ± 0.10	*	0.03 ± 0.004	0.05 ± 0.01	*
LCN6-Ph_{30}	0.13 ± 0.006	0.21 ± 0.006	*	0.07 ± 0.006	0.14± 0.005	*

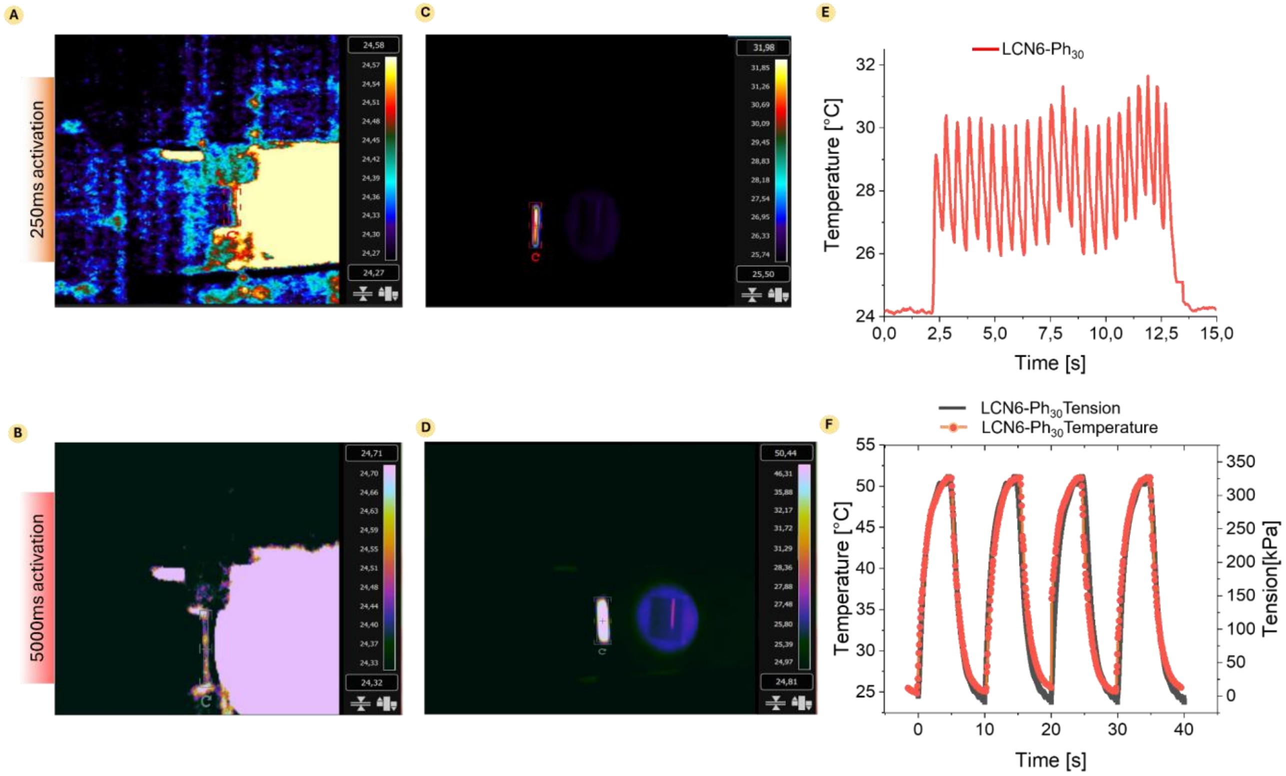

Figure S22: Thermal characterization of a LCN6-Ph$_{30}$ film during irradiation. A, B) Surface temperature before light-activation. C, D) Maximum temperature reached by the sample under 470 nm illumination with two different activation times: 250 ms (C) and 5000 ms (D); E) Real-time temperature trace of the sample under multiple cycle of activation for 250 ms; F) Real-time temperature trace of the sample under multiple cycle of activation for 5000 ms. The graph also includes the comparison with the light-tension development trace. Surface temperature of the films was acquired during the photo-activation through a region of interest (sample dimensions). Irradiation intensity reported in the graphs was 4.4 mW/mm^2.

Table S9: Thermal characterization of LCNs for short activation times. T: average and standard deviation of the maximum surface temperature reached by sample. On the left of the table is reported the effect of the lateral spacer while on the right the effect of the crosslinker content**.** The reported data are an average of 10 different activations and relaxations for three strips taken from three different LCN films. Irradiation intensity was varied according to the tension development tests: 2.3 mW/mm^2, 4.4 mW/mm^2 and 6.2 mW/mm^2.

**LCN | 250 ms**	T [°C] 2.3 mW/mm^2	T [°C] 4.4 mW/mm^2	T [°C] 6.2 mW/mm^2	**LCN | 250 ms**	T [°C] 2.3 mW/mm^2	T [°C] 4.4 mW/mm^2	T [°C] 6.2 mW/mm^2
LCN3-Ph_{10}	28 ± 0.6	32 ± 0.7	34 ± 2.6	LCN6-Ph_{10}	30 ± 0.6	32 ± 0.9	34 ± 2.8
LCN6-Ph_{10}	30 ± 0.6	32 ± 0.9	36 ± 1.2	LCN6-Ph_{20}	28 ± 1	31 ± 1.4	32± 2
LCN9-Ph_{10}	29 ± 0.8	33 ± 1.6	37 ± 1.9	LCN6-Ph_{30}	28 ± 1.5	31 ± 2	33 ± 2
